# Comparative Morphology of Premolar Foramen in Lagomorphs (Mammalia: Glires) and Its Functional and Phylogenetic Implications

**DOI:** 10.1371/journal.pone.0079794

**Published:** 2013-11-21

**Authors:** Łucja Fostowicz-Frelik, Jin Meng

**Affiliations:** Division of Paleontology, American Museum of Natural History, New York, New York, United States of America; Raymond M. Alf Museum of Paleontology, United States of America

## Abstract

Lagomorphs (a group that consists of pikas, hares, rabbits and allies) are notable for their conservative morphology retained for most of their over 50 million years evolutionary history. On the other hand, their remarkable morphological uniformity partly stems from a considerable number of homoplasies in cranial and dental structures that hamper phylogenetic analyses. The premolar foramen, an opening in the palate of lagomorphs, has been characterized as an important synapomorphy of one clade, Ochotonidae (pikas). Within Lagomorpha, however, its phylogenetic distribution is much wider, the foramen being present not only in all ochotonids but also in leporids and stem taxa; its morphology and incidence also varies considerably across the order, even intraspecifically. In this study, we provide a broad survey of the taxonomic distribution of the premolar foramen in extant and fossil Lagomorpha and describe in detail the morphological variation of this character within the group. Micro-computed tomography was used to examine the hard palate and infraorbital groove morphology in *Poelagus* (Leporidae) and *Ochotona*. Scans revealed the course and contacts of the canal behind the premolar foramen and structural differences between the two crown clades. We propose that the premolar foramen has evolved independently in several lineages of Lagomorpha, and we discuss development and function of this foramen in the lagomorph skull. This study shows the importance of comprehensive studies on phylogenetically informative non-dental characters in Lagomorpha.

## Introduction

Lagomorpha is a mammalian order [1] and clade [2] known from the Eocene to Recent of Asia and North America, the Oligocene to Recent of Europe, the Miocene to Recent of Africa and the Pliocene to Recent of South America [1]. The clade is well known for many unresolved evolutionary relationships above the species level, in particular among its fossil representatives. Lagomorphs are generally considered to be the sister clade to Rodentia in the cohort Glires and originated in Asia, where their earliest fossil representatives are known from the Early Eocene of China [3], India [4], Kyrgyzstan [5] and Mongolia [6].

The premolar foramen was named (as *foramen premolare*) and described by Bohlin [7] who found it in the skulls of extant *Ochotona*, and Oligocene *Desmatolagus gobiensis* and *Sinolagomys kansuensis*, (Bohlin [7]: 58–60). The presence of the premolar foramen has been used since then as a phylogenetic character in lagomorph systematics (as a diagnostic feature for ochotonids; e.g., [7]–[11]), but other studies show that the premolar foramen is also present in some undoubtedly leporid species (e.g., [12]–[15]). Further, the status of the premolar foramen as an ochotonid synapomorphy is put in doubt by its presence in some species of *Desmatolagus* [16]. Some authors assigned this speciose and diverse genus to the Ochotonidae [1], [7], [17], while others placed it in the Leporidae [16], [18]–[20]. However, we consider *Desmatolagus* a stem group that is most probably paraphyletic.

In this paper we do not propose a definitive lagomorph phylogeny; our approach is preliminary, focused on morphology and incorporates our new results (as regards extinct lineages) and available phylogenetic analyses [21]–[26]. We recognize major nodes within the Duplicidentata following Asher et al. [24]. The crown Lagomorpha consists of all extant lineages (pikas, hares and rabbits), their most recent common ancestor and all its fossil descendants. Stem lagomorphs are taxa more closely related to crown Lagomorpha than to *Gomphos*.

Although lagomorphs are often studied in taxonomic and ecological contexts, anatomical studies on this group are rare. Quite recently, Bleefeld and Bock [27] described the calcaneal canal as a synapomorphy of the order, which had escaped notice of earlier researchers probably due to its minute size. The prevalence of dental remains in Lagomorpha brought about the overreliance on certain characters of P2 and p3 and led to neglect of cranial and postcranial anatomy. This specialized approach is deficient and points to the importance of studies based on comparative anatomy for making phylogenetic inferences and for interpreting functional morphology in Lagomorpha. Therefore, we present the first thorough examination of current knowledge on taxonomic distribution of the premolar foramen across extant and extinct lagomorphs, on its incidence and structure, and we discuss its evolution and phylogenetic significance. We also hypothesize on its functional meaning.

## Materials and Methods

We gathered data both on extant (all the genera, 12 in total) and fossil taxa. In the case of the extinct species, cranial material was studied by us and additional data were provided by published descriptions. We included as many individuals of each species as possible to account for variation. Understandably, we omitted numerous fossil taxa that lack a well-preserved portion of the palate. For example, there is no cranial material of *Arnebolagus*, *Gripholagomys*, *Hypsimylus*, and *Tachylagus* (stem lagomorphs), *Albertona*, *Bellatonoides*, *Marcuinomys* and *Paludotona* (ochotonids), or *Aluralagus*, *Aztlanolagus*, *Paranotolagus* and *Pronotolagus* (leporids) for us to make any inference on the premolar foramen. Also, within a genus, sometimes we have data on only some species (e.g., one of *Alloptox*, or two of *Archaeolagus*). To examine the patency and course of a canal behind the premolar foramen, the opening in extant specimens was probed with a thin two-core copper wire (a single filament was 0.12 mm in diameter).

Micro-CT imaging of the skulls of *Poelagus* (a leporid) and *Ochotona* was performed with a Phoenix v|tome|x L 240 scanner (GE Measurement & Control Solutions) with the following parameters: voltage 130 (145) kV, current 160 (220) µA, and no filter (0.1 mm Cu filter), respectively. To accommodate for length of the specimens, two scans of each skull (multiscans) were performed. In the first case, images were acquired at an isotropic resolution of 22 µm (cubic voxels) with 4600 (2300 per region) views, with 0.33 sec of exposure. For *Ochotona*, images were acquired at a resolution of 24 µm (cubic voxels) with 4000 (2000 per region) views, with 0.33 sec of exposure. Raw data were reconstructed with Phoenix datos|x 2.0 software. Reconstructed-scan data were viewed and analyzed with Volume Graphics VG Studio Max 2.1.

**Figure 1:**
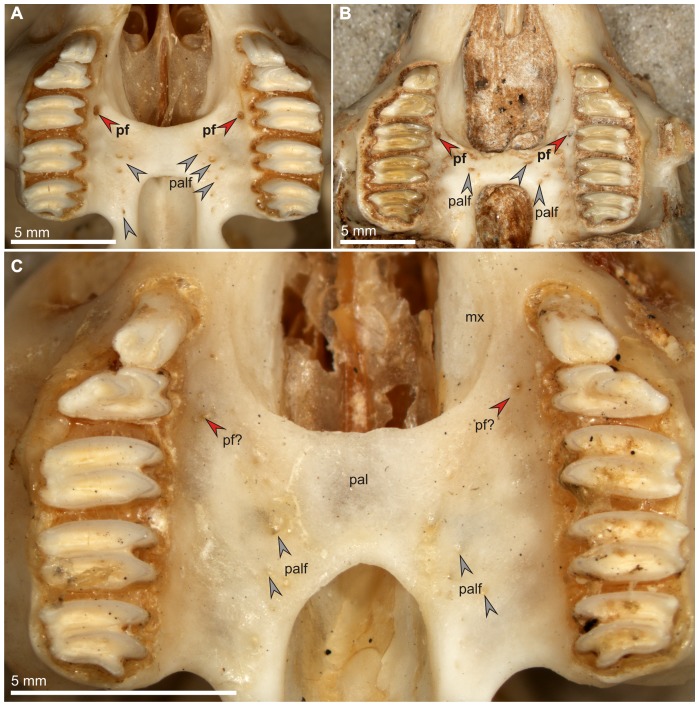
Palate structure and appearance of the premolar foramen in modern and fossil *Ochotona*. A. *Ochotona princeps*, (AMNH 32717), Recent, USA, both premolar foramina well-developed, medial to P4. B. *Ochotona chowmincheni*, (AMNH F:AM 141394, holotype), Late Miocene, Shanxi, China, both premolar foramina well-developed, medial to P4. C. *Ochotona collaris*, (AMNH 137268), Recent, Canada, with poorly developed rudimentary foramina. Abbreviations: mx, maxilla; pal, palatine; palf, palatine foramina; pf, premolar foramen.

In order to trace evolution of the premolar foramen in lagomorph lineages, we constructed the morphological character matrix (Dataset S1) that includes 80 cranial and dental characters (see List S1). Overall, we included 22 taxa in our data set, which represent all major groups of living and fossil Lagomorpha; the latter were selected on the basis of completeness of morphological characters. Outgroup is *Mimotona wana*, a Paleocene Asian mimotonid. Phylogenetic analyses were performed using PAUP* v. 4.0b10 [28] and Mesquite v. 2.75 [29]. The photographs in Figs. 1, 3, 4, 5, 9, 11A, B, 12, 13 were taken with a Nikon DS-Fi1 camera attached to Nikon SMZ-U binocular microscope as a Z-stack and merged into a single photograph with Helicon Focus 5.1 software; the photographs in Fig. 10 were combined from the stack shots using Helicon Focus software.

**Figure 2:**
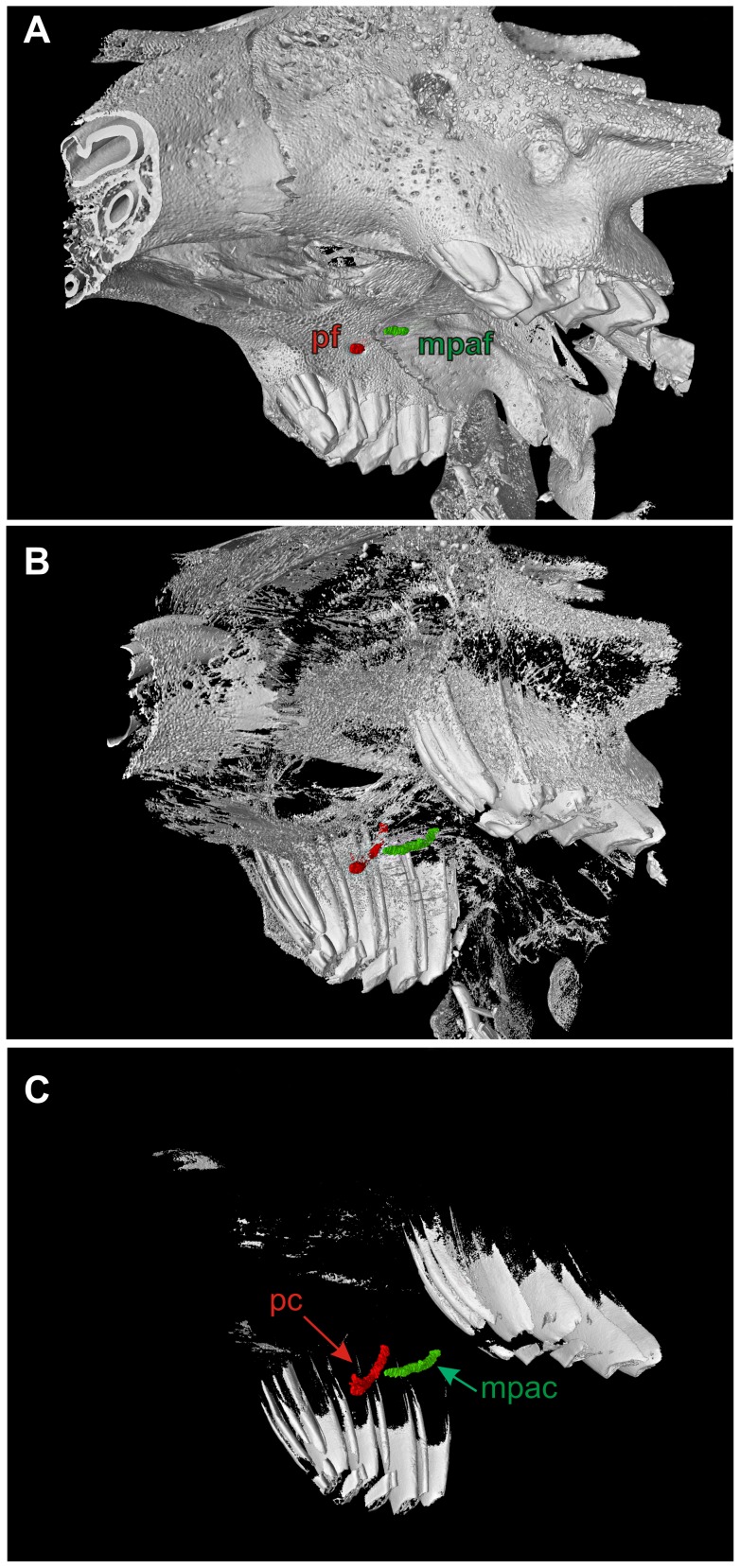
Reconstructed course of main canals in the hard palate of *Poelagus marjorita* (AMNH 51183). Premolar (in red) and palatine (in green) canals; based on μCT images. A–C. Gradual digital elimination of bone tissue enabled visualization of the entire course of both canals. Abbreviations: mpac, major palatine canal; mpaf, major palatine foramen; pc, premolar canal; pf, premolar foramen.

**Figure 3:**
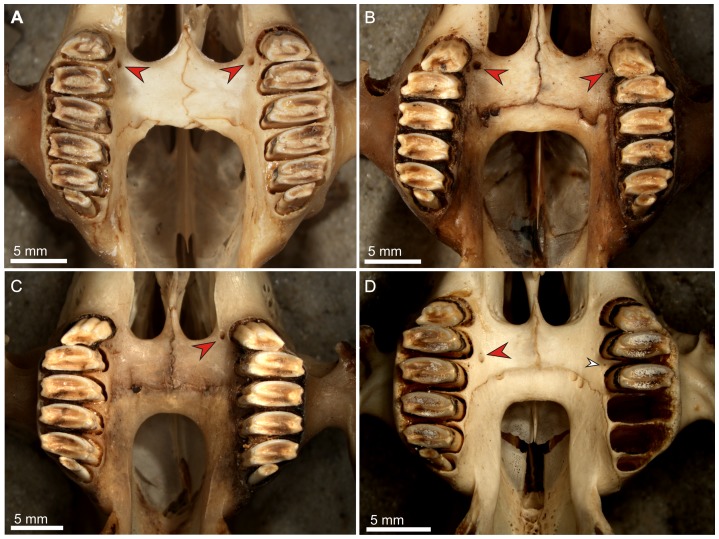
Palate structure and appearance of the premolar foramen in extant *Lepus*. A. *L. callotis* (AMNH 35158), a pair of premolar foramina placed symmetrically medial to the P2/P3 alveolar septum. B. *L. oiostolus* (AMNH 113705), showing asymmetrically developed foramina at the P2 alveolus (the left foramen is rudimentary). C. *L. microtis* (AMNH 80834), single foramen located at the left side, medial to the P2 alveolus. D. *L. nigricollis* (AMNH 102449) with asymmetrically developed premolar foramen at the distal margin of the P3 alveolus (right, red arrow) and P3/P4 alveolar septum (left, accessory foramen, white arrow).

**Figure 4:**
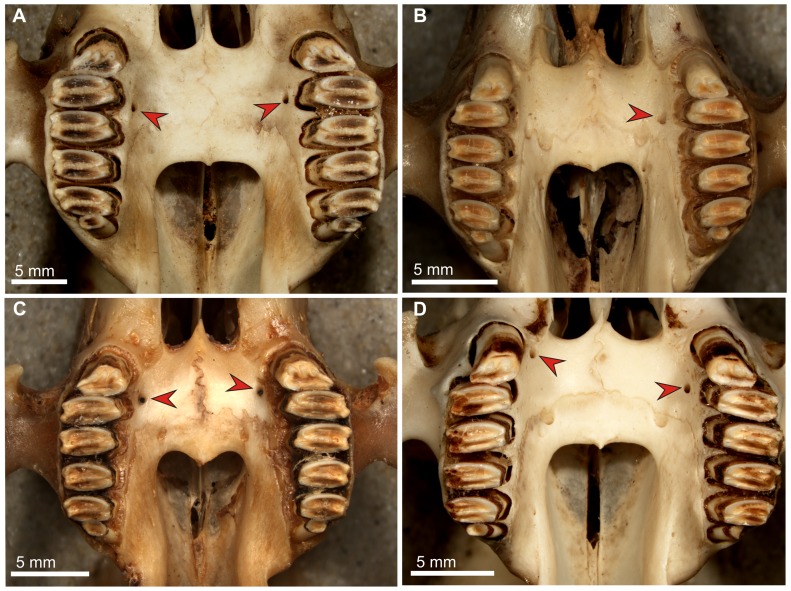
Palate structure and appearance of the premolar foramen in *Sylvilagus*. A. *Sylvilagus aquaticus* (AMNH 2223), large paired foramina (arrows) typical for this species, placed slightly asymmetrically at the P3 alveolus. B. *S. floridanus* (AMNH 7408), note a single large foramen located medially to the P3 alveolus. C. *S. brasiliensis* (AMNH 66663) showing a pair of large foramina placed slightly asymmetrically, medial to the P2/P3 alveolar septum. D. *S. palustris* (AMNH 256779), asymmetric arrangement of foramina at the P2 (right) and P3 (left) alveoli.

**Figure 5:**
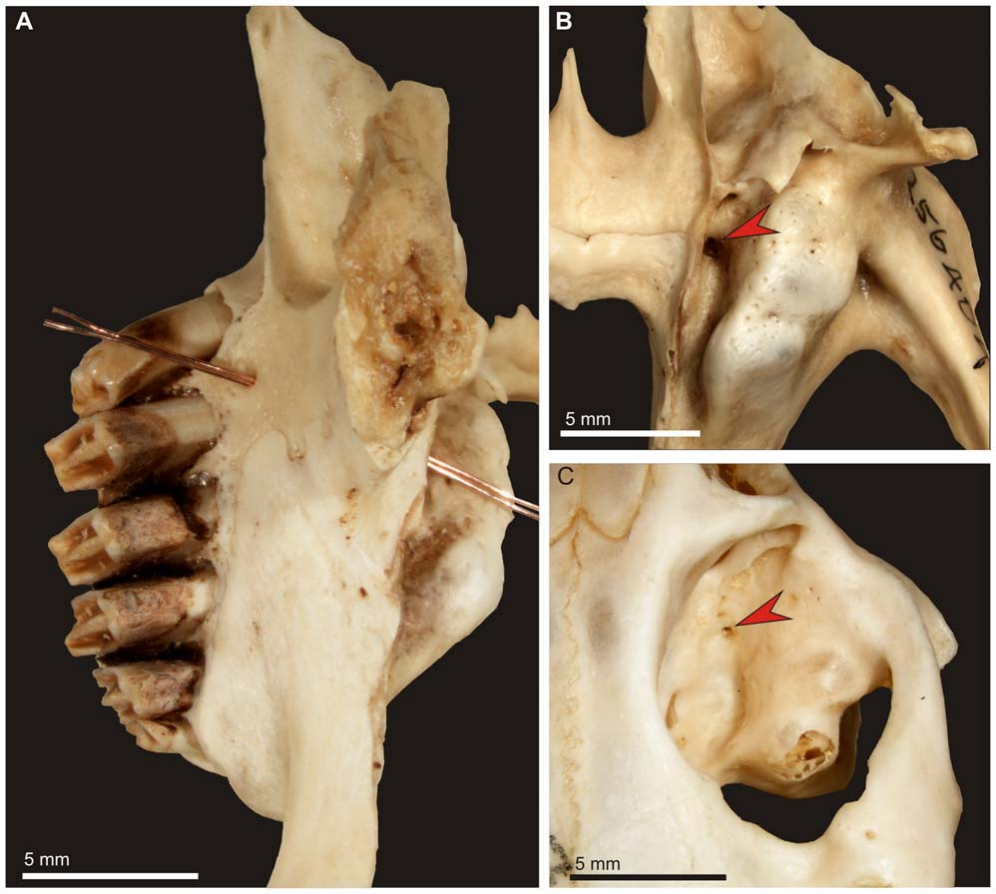
Morphology of the premolar canal in extant leporids and ochotonids. A. *Sylvilagus palustris hefneri* (AMNH 256404), right maxilla and palatine in lingual view; wire shows an inclined course of the canal. B. The same, note large dorsal opening of the premolar canal in the infraorbital groove (arrow). C. Dorsal entrance to the premolar canal in *Ochotona princeps saxatilis* (AMNH 32717), note relatively small diameter of the canal.

**Figure 6:**
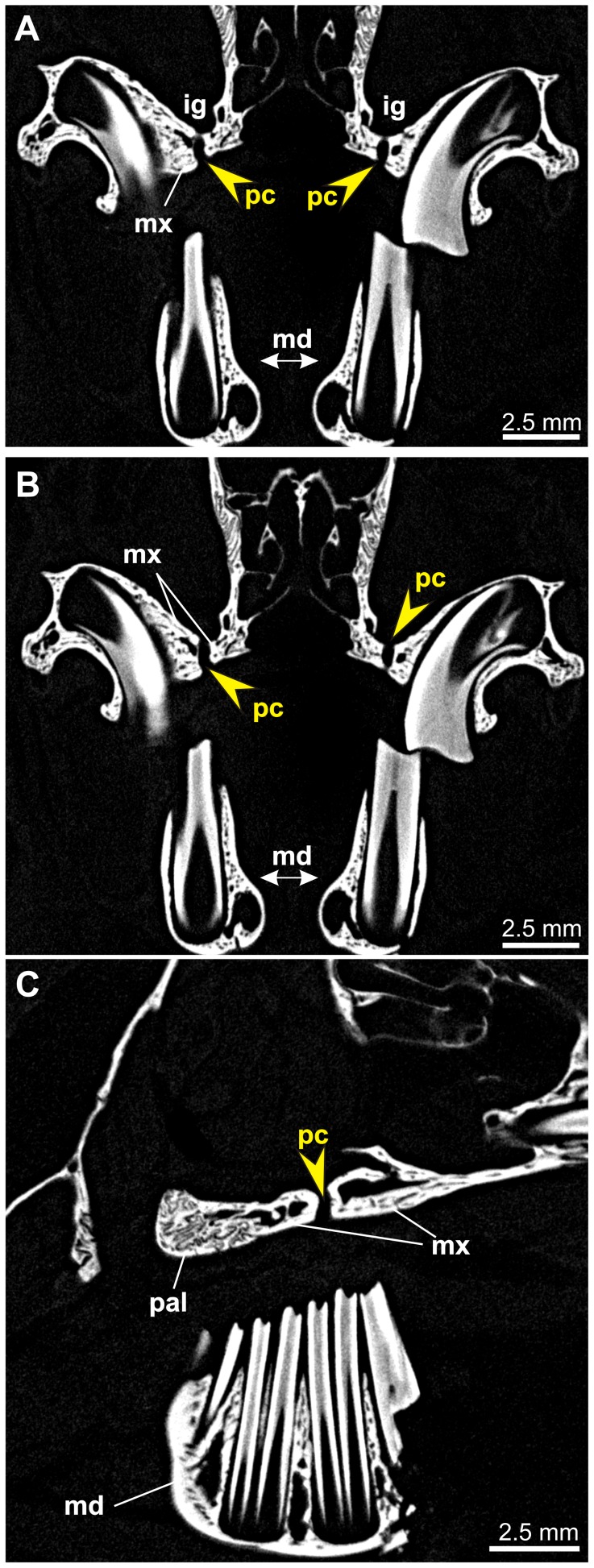
Micro-CT images of the hard palate in *Ochotona princeps* (AMNH 95203). Sections through the palate region in frontal (A and B) and sagittal (C) planes, showing a short and relatively vertical premolar canal (pc, arrows). Note wide and shallow infraorbital groove (ig). Other abbreviations: md, mandible; mx, maxilla; pal, palatine.

**Figure 7:**
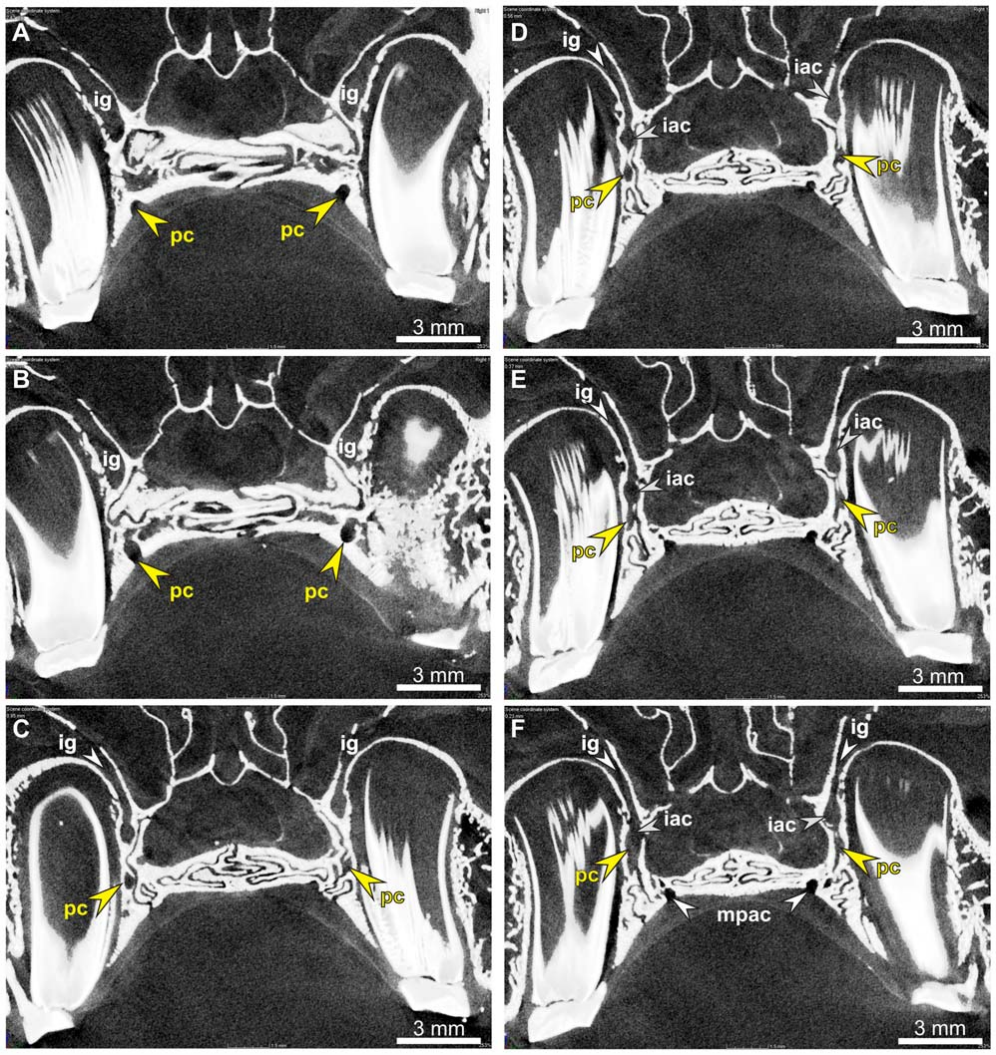
Micro-CT images of the hard palate in *Poelagus marjorita* (AMNH 51183). A–F. Sequence of sections in frontal plan of the hard palate area (A, frontalmost to F, caudalmost) show the course of the premolar canal (pc, arrows) and the structure of the extremely narrow infraorbital groove (ig). The premolar canal goes along the maxillo-palatine suture. Note a canal for the infraorbital artery (iac; C–F) that merges with the premolar canal (E, left side, F, both sides). Abbreviation: mpac, major palatine canal.

**Figure 8:**
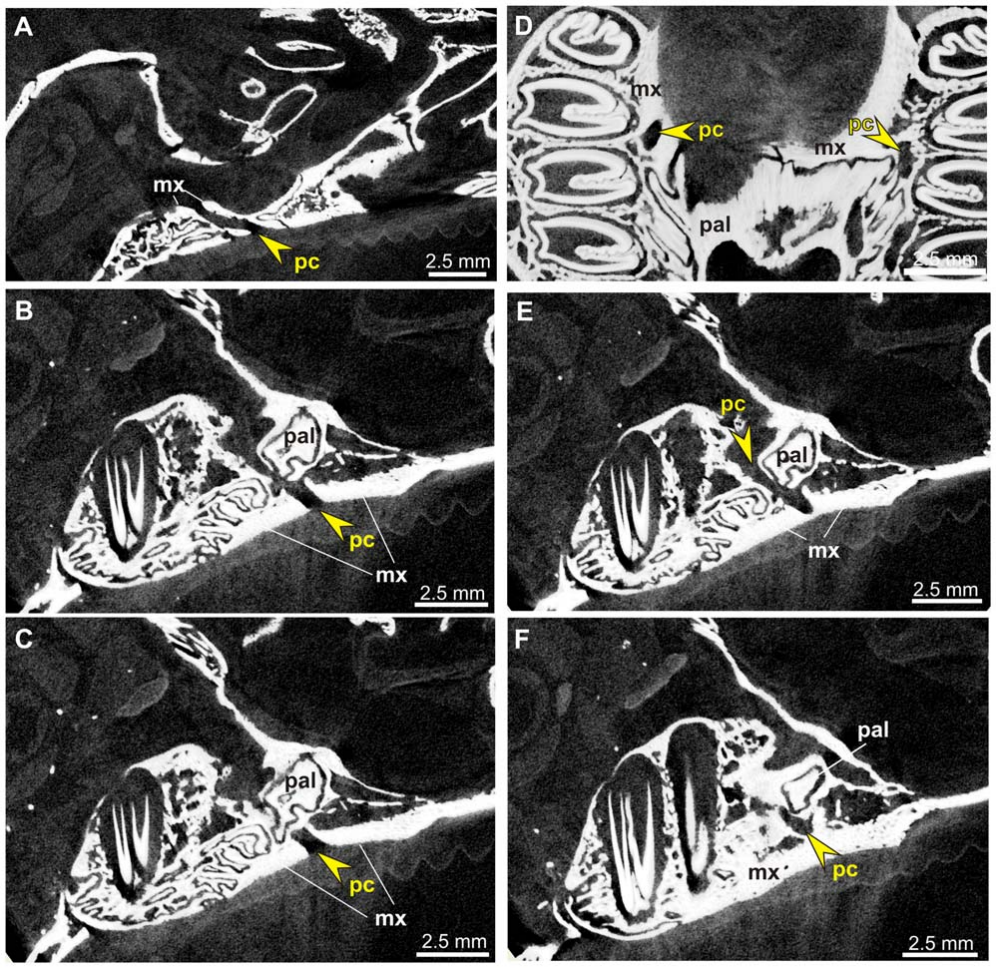
Micro-CT images of the hard palate in *Poelagus marjorita* (AMNH 51183). A–C, and E–F. Sequence of sections in sagittal plane (A, medialmost to F, buccalmost) through the palatine process of the maxilla which hosts the premolar canal (pc, arrow); note complicated route of the maxillo-palatine suture. D. Section in palate plane shows well-defined premolar foramina (arrows) close to the maxillo-palatine suture. Abbreviations: mx, maxilla, pal, palatine.

**Figure 9:**
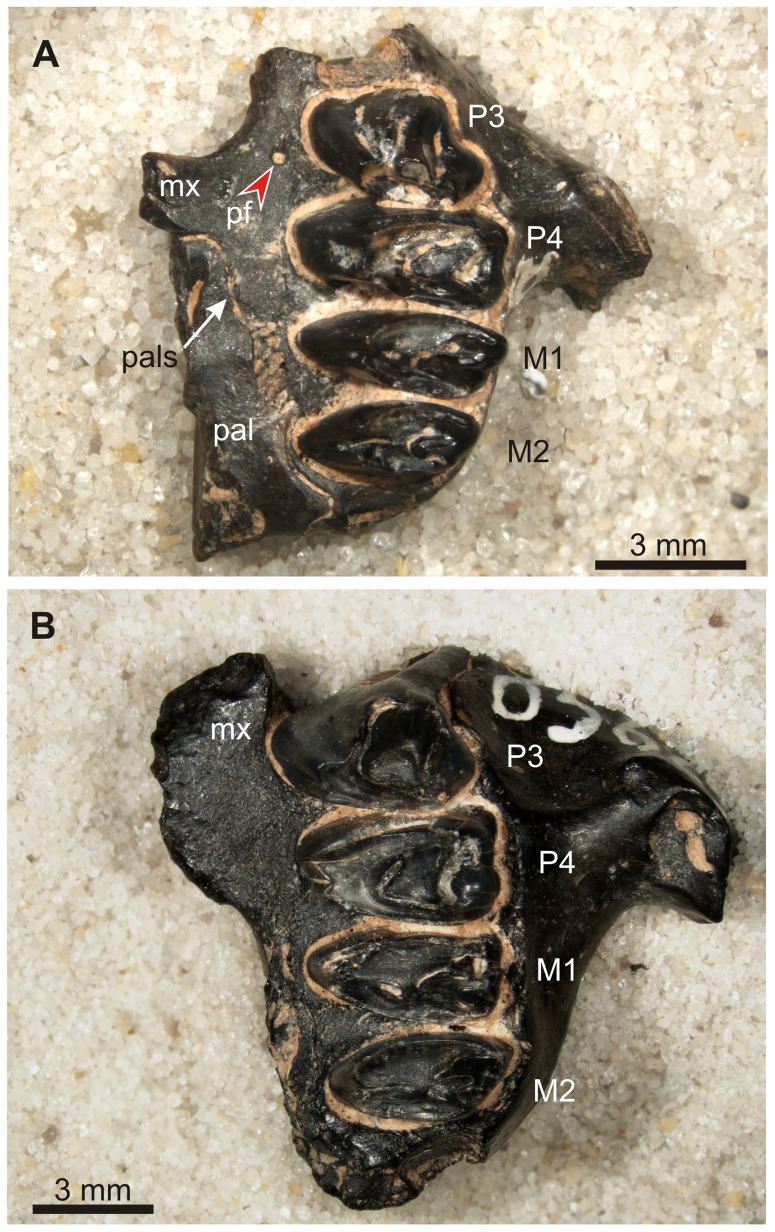
Premolar foramen in *Desmatolagus*. A. *Desmatolagus gobiensis* (AMNH FM 85072), Tsagan Nor Basin, Mongolia (Oligocene), left maxilla with P3–M2, note a small premolar foramen medial to the distal part of the P3 alveolus. B. *Desmatolagus robustus* (AMNH FM 85666), Tsagan Nor Basin, Mongolia (Oligocene), left maxilla with P3–M2; note lack of the premolar foramen. Abbreviations: mx, maxilla; pal, palatine; pals, maxillo-palatine suture; pf, premolar foramen.

**Figure 10:**
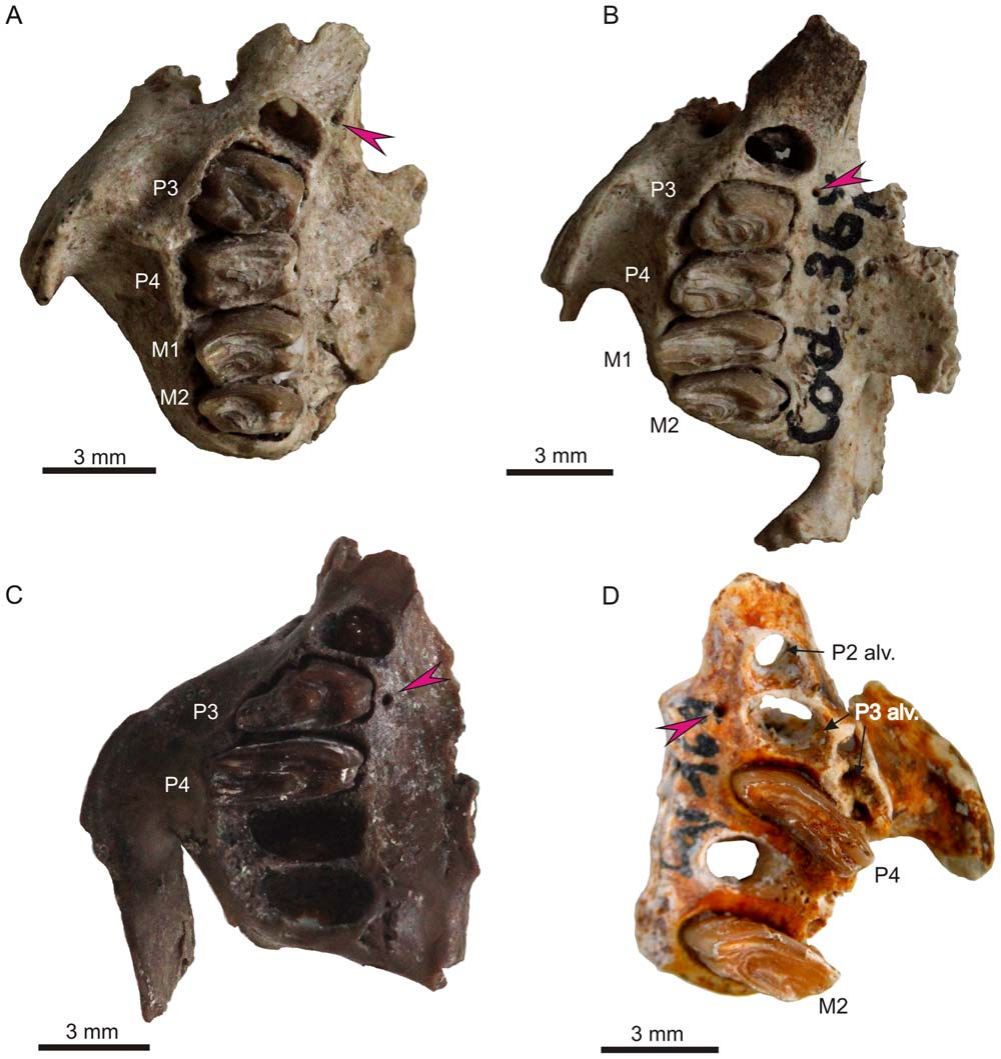
Premolar foramen in European stem taxa. A. *Amphilagus antiquus* (NMB Bst. 226), note small premolar foramen at the antero-medial margin of P2 (arrow). B. *Piezodus branssatensis* (NMB Cod. 367). C. *Titanomys visenoviensis* (NMB M.A. 7626). D. *Eurolagu*s *fontannesi* (ML Lgr. 169, type specimen). A–C, Oligocene, Coderet-Bransat, Allier, France; D, Middle Miocene, La Grive, Isère, France.

**Figure 11:**
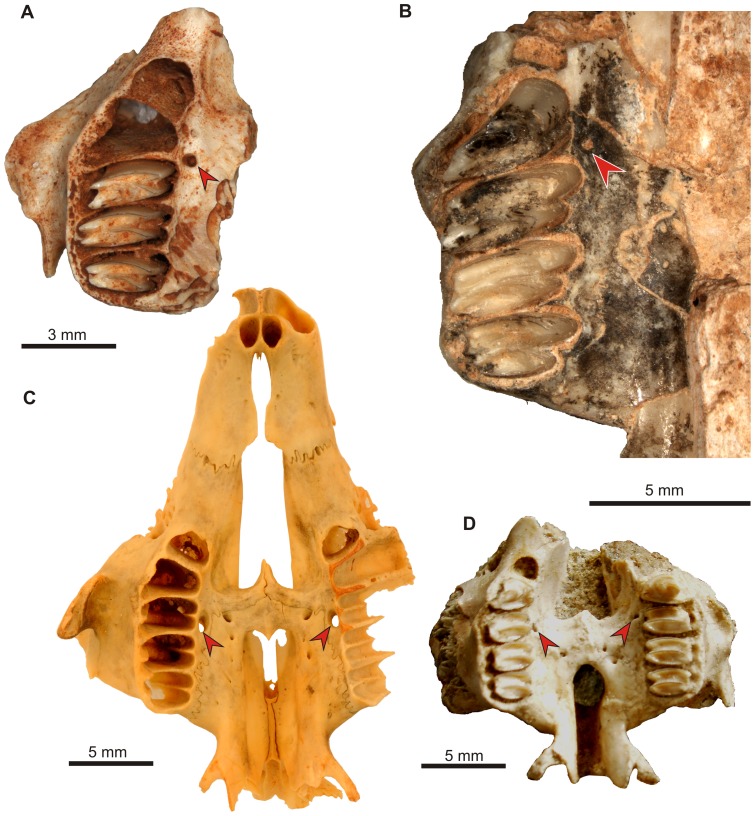
Palate structure and appearance of the premolar foramen in prolagids and extinct ochotonids. A. *Prolagus oeningensis* (AMNH FM 10519), La Grive St. Alban, Isère, France (Miocene), right maxilla with P4–M2, the premolar foramen between P3 and P4 alveoli. B. *Sinolagomys* sp. (AMNH uncataloged), Tatal Gol, Mongolia (Oligocene), skull fragment with right maxilla with P3–M2 and the premolar foramen medial to the distal part of the P3. C. *Prolagus sardus* (CM uncataloged), Corbedu Cave, Sardinia (Pleistocene), skull fragment. Note the enlargement and backward shift of the premolar foramen (at the P4 alveolus). D. *Alloptox gobiensis* (IVPP V 13054.8, figured in part), Middle Miocene, Ningxia Hui, China.

**Figure 12:**
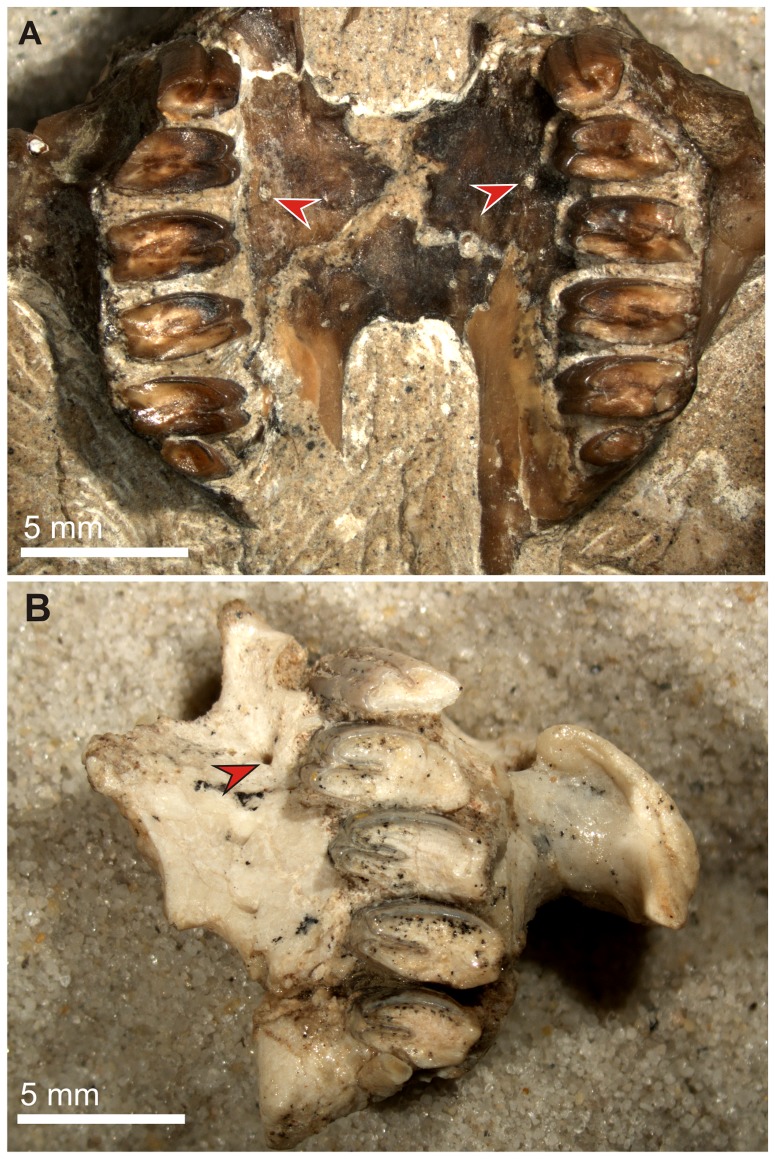
Palate structure and appearance of the premolar foramen in *Archaeolagus* (Leporidae). A. *Archaeolagus ennisianus* (AMNH FM 7190, holotype), John Day Formation, Oregon (Arikareean), paired foramina medial to the distal margin of the P3 alveoli. B. *Archaeolagus* cf. *macrocephalus* (AMNH F:AM 22292), Standing Rock Q, Sandoval Co. New Mexico (?Arikareean/Hemingfordian), small foramen medial to the P3 alveolus, left maxilla with P3–M2 preserved.

**Figure 13:**
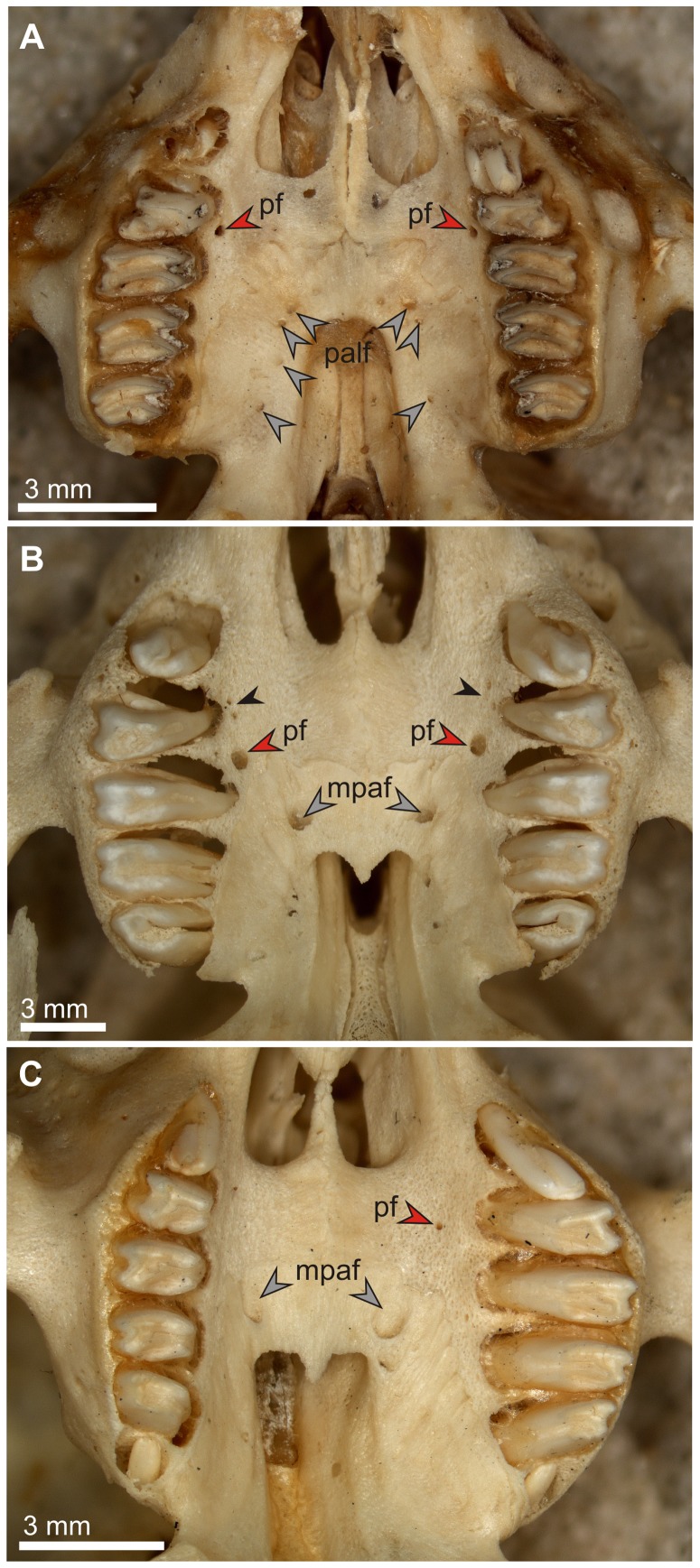
Palate structure and condition of the premolar foramen in juvenile extant lagomorphs. A. *Ochotona pallasi* (AMNH 59780), note well-developed premolar foramina (pf, red arrows) medial to the DP3/DP4 septum, and small multiple palatine foramina (palf, grey arrows) characteristic for *Ochotona*. B. *Sylvilagus palustris* (AMNH 253183) with well-developed premolar foramina (pf, red arrows) medial to the DP3/DP4 septum, matching in size the major palatine foramina (mpaf, grey arrows), note minute accessory nutritive foramina near the DP3 (small black arrows) and a slight asymmetry in the position of premolar foramina. C. *Brachylagus idahoensis* (AMNH 140865), note a minute foramen medial to the DP3 (pf, red arrow) and a pair of large palatine foramina (mpaf, grey arrows).

### Permission and Institutional abbreviations

This work is based on museum specimens only. In compliance with all relevant regulations we examined specimens at the following institutions: AMNH, American Museum of Natural History, New York, USA; ANSP, Academy of Natural Sciences of Philadelphia, Philadelphia, USA; BMNH, British Museum of Natural History, London, UK; CM, Carnegie Museum of Natural History, Pittsburgh, USA; ZMB, Museum für Naturkunde, Berlin, Germany. Additional data were supplied by IMNH, Idaho Museum of Natural History, Pocatello, USA; IVPP, Institute of Vertebrate Paleontology and Paleoanthropology, Beijing, China; ML, Musée des Confluences, Lyon, France; NMB, Naturhistorisches Museum, Basel, Switzerland. No specimens were destructively sampled or purchased.

## Results

### Morphology of the premolar foramen

The premolar foramen is a small round or oval opening, varying from ca. 0.25 mm to over 1.5 mm in diameter, located in the palatine process of the maxilla lingually and close to the premolar alveoli (Fig. 1). It mainly occurs at the P3 or P4 alveolus, but it can be found at the entire area between P2 and the anterior margin of M1, including the transition area between alveoli. The premolar foramen should not be mistaken for the palatine foramina (Figs. 2, 3, 4), which mainly occur more distally, either at the maxillo-palatine suture (Figs. 3, 4A, B, D) or within the palatine portion (Figs. 1, 4C) of the hard palate [15], [30] and are known in most of crown mammalian clades (e.g., posterior palatine foramina in rodents; see [31]).

Most often, the paired premolar foramina occur symmetrically on the palate (Figs. 1, 3A, 4A, C). However, the incidence and development of the premolar foramen are highly variable. The foramen can be present only on one side (Fig. 3C) or it can be well-developed on one side, but greatly reduced on the other (Fig. 3B, D). Further, the position can be asymmetric, one of the pair being placed more anteriorly than the other (Fig. 4D).

Apart from the proper premolar foramen that leads into the fully functional canal (see further) also some ‘accessory foramina’ may occur at the palatine process of the maxilla. They are either single or serial minute pits (Fig. 3D). Most probably, they serve as openings for local nutritive blood vessels for the palate mucous membrane and periosteum.

### Premolar canal

The premolar foramen is the ventral opening of a small canal (we propose to term it the premolar canal) piercing the maxilla and leading into the orbit (Figs. 2, 5, 6, 7, 8). The premolar canal goes slightly obliquely in distal direction and enters the orbit floor ventrally, in the infraorbital groove (Figs. 5B, C, 6A–B, 7). In all cases the premolar canal opens dorsally into the infraorbital groove. The exact morphology of the premolar canal in Lagomorpha reflects the structure of the infraorbital groove which differs markedly between Leporidae (Figs. 5B, 7) and Ochotonidae (Figs. 5C, 8).

Ochotonids and prolagids have shallow and wide infraorbital groove, created by lower and more laterally reclined alveolar processes. The structure of the infraorbital groove in ochotonids can be described as ‘open’ (Figs. 5C, 7), in opposition to the deep and narrow (‘closed’) infraorbital groove in leporids. The latter is created by higher alveolar process of the maxilla, laying closely to the nasal surface of the body of the maxilla (Figs. 5B, 7). The orbital aperture of the premolar canal is well-visible in the floor of the orbit in *Ochotona* (Fig. 5C), while in leporids it is concealed in the infraorbital groove between the alveolar sockets and the wall of the orbit (Fig. 5B).

High resolution CT-scans of the skulls of *Ochotona princeps* (Fig. 6) and *Poelagus marjorita* (Figs. 2, 7, 8) allowed us to reconstruct the topography and orientation of the premolar canal. The differences lie in length and exact position of the canal within the hard palate. The premolar canal in *Ochotona* is shorter and has more vertical course (Fig. 6) than in *Poelagus* (Figs. 2, 8); moreover, it is entirely hosted by the maxilla and its distance to the maxillary-palatine suture is noticeable (Fig. 6). On the other hand, in *Poelagus* the canal runs along the maxillo-palatine suture and is in part formed by the palatine (Fig. 7C–F), although most of its course runs only within the maxilla. Furthermore, the premolar canal in *Ochotona* has smooth walls, whereas in *Poelagus* it contacts at some points with the alveoli (Fig. 7B, D) by thin and irregular isthmuses.

### Distribution

The premolar foramen is relatively widely distributed across lagomorph taxa and can be found in representatives of both Ochotonidae and Leporidae as well as some stem groups of Lagomorpha (Figs. 9, 10). Overall, it is less common in Leporidae, whereas its presence was confirmed in all the genera of Ochotonidae for which the cranial material is known. The premolar foramen is absent in rodents (see [31] and eurymylids, e.g., *Eurymylus* [32] and *Rhombomylus* [2]).

#### Mimotonidae and stem Lagomorpha

The premolar foramen is absent in Mimotonidae, a paraphyletic group of stem Duplicidentata, within which monophyletic Lagomorpha is nested [24], [33], [34]. It was not reported from *Gomphos*, *Mimolagus*, and *Mimotona* [15], [24], [35]–[37]. On the other hand, many taxa regarded as stem lagomorphs, a heterogeneous group outside (Prolagidae+Ochotonidae)+Leporidae clade [24], [33], show variability in the presence and development of the premolar foramen. The premolar foramen has not been detected in most of the oldest representatives of Lagomorpha from Asia, such as *Dawsonolagus* [3], *Lushilagus* [38], *Shamolagus* [38], and *Strenulagus* [39]. Also several Eocene and Oligocene North American taxa referred to stem lagomorphs [40] lack the foramen; these include *Chadrolagus emryi*, all species of *Mytonolagus*, *Megalagus* (*M. brachyodon* and *M. turgidus*), all species of *Palaeolagus* with known palate portion (*P. burkei*, *P. haydeni*, *P. hypsodus*, *P. intermedius*, *P. philoi*, and *P. temnodon*), and ‘*Procaprolagus*’ *vusillus* [9], [29], [41]–[46]. Table 1 presents stem taxa with known occurrence of the premolar foramen (see also Figs. 9, 10 for *Desmatolagus* and some European taxa).

**Table 1:** Incidence of the premolar foramen in stem lagomorphs.

Genus	Presence	Comments	References
*Aktashmys*	**+** in *A. montealbus*	A well-defined premolar foramen is placed medially to the P4.	[5]: fig. 2c
*Amphilagus*	**+** in *A. antiquus*; **?** in ‘*A.*’ *ulmensis*, ‘A’. *sarmaticus*, and *A. wuttkei*	Variably placed; a small premolar foramen medial to the P2/P3 interalveolar septum was documented by Viret [68]. Specimen NMB Bst. 226 from Coderet-Branssat (France) has a small premolar foramen at the antero-medial margin of the P2 alveolus (Fig. 10A).	[68]: pl. XXIX: 10 a, b; [69]–[71]
*Desmatolagus*	**+** in *D. gobiensis*, *D. periaralicus*, *D. simplex*; **−** in *D. ardynensis* 1, *D. robustus*, *D. vetustus*; **∼** in *D. pusillus*; **?** in *D. kazachstanicus*, *D. moergenensis*, *D. veletus*	The premolar foramen medial to the P3 alveolus or in the area between P3 and P4 (Fig. 9A). In *D. pusillus* Huang [64] reported very few individuals completely lacking this foramen.	[7]: fig. 17a, b; [16], [18], [19]; [63]: fig. 5; [72]–[74]
*Eurolagus*	**+** in *E. fontannesi*	A relatively small premolar foramen is medial to the P3 in holotype ML LGr 169 (Fig. 10D).	[75]: pl. 13: figs. 19-19b
*Gobiolagus*	**+** in *G. lii*; **−** in *G. aliwusuensis*, *G. tolmachovi*; **?** in *G. andrewsi*, *G. burkei*, *G. major*	A small premolar foramen occurs in the holotype and only specimen of *G. lii* (IVPP V 12755). All specimens of *G. tolmachovi* lack premolar foramen, apart from IVPP V8430 where it occurs medial to P3 (see [16] for discussion).	[16]; [59]: fig. 1; [60]: fig. 1; [63]; [76]
*Piezodus*	**+** in *P. branssatensis*; **?** in *P. tomerdingensis*	A small premolar foramen is placed medially to the P2/P3 interalveolar septum or more closely to the P3 alveolus in *P. branssatensis* (Fig. 10B).	[68]: pl. XXIX, fig. 15 b; [77]
*Titanomys*	**+** in *T. visenoviensis*; **?** in *T. calmaensis*	A small foramen medial to the P3 (Fig. 10C).	[7]: 60; [68]: pl. I, fig. 22; [69]

Symbols: ‘**+**’, foramen present in all examined specimens; ‘**−**’, absent in all examined specimens, ‘**?**’ not known, ‘**∼**’ variable.

1 Spelling corrected from “*ardynense*” under Article 31.2 of the ICZN [78].

#### Ochotonidae and Prolagidae

Overall, Ochotonidae and closely related Prolagidae (Fig. 11) have a pair of premolar foramina placed not further back than the distal margin of the P4 alveolus [7], [9]. We examined the morphology and location of the premolar foramen in the fossil and extant representatives; however, our comments on the variability of this character are limited mainly to extant *Ochotona* (Figs. 1, 5C, 6). Table 2 summarizes data on both families.

**Table 2:** Incidence of premolar foramen in fossil ochotonids and prolagids.

Genus	Presence	Comments	References
*Alloptox*	**+** in *A. gobiensis*	A well-developed premolar foramen is medial to the alveolar region between P3 and P4 or P4 (Fig. 11D).	[79]: 1b
*Bellatona*	**+** in *Bellatona forsythmajori*	A well-defined premolar foramen is medial to the P4 alveolus.	[80]: fig. 4
*Hesperolagomys*	**+** in *H. galbreathi*, *H. niobrarensis*; **?** in *H. fluviatilis*	In *H. galbreathi* and *H. niobrarensis* the premolar foramen is mostly medial to the distal margin of the P3 alveolus.	[11]: figs 3, 9; [81]
*Kenyalagomys*	**+** in *K. rusingae*; **?** in *K. minor*	The premolar foramen is medial to the P3/P4 interalveolar septum in *K. rusingae*.	[82]: fig. 2
*Ochotonoides*	**+** in *O. complicidens*; **?** in *O. primitivus*		[48]
*Oreolagus*	**+** in *O. nebrascensis*, *O. nevadensis*, *O. wallacei*, *O. wilsoni*	Premolar foramen is medial to the P3 in *O. nebrascensis*, *O. nevadensis*, and *O. wilsoni*, and medial to the P3/P4 interalveolar septum in *O. wallacei*.	[65]; [83]: figs. 1, 30
*Prolagus*	**+** in *P. apricenicus*, *P. calpensis*, *P. crusafonti*, *P. figaro*, *P. ibericus*, *P. imperialis*, *P. italicus*, *P. major*, *P. michauxi*, *P. oeningensis*, *P. sansaniensis*, *P. sardus*, *P. sorbini*, *P. tobieni*, *P. vasconiensis*; **?** *P. bilobus*, *P. caucasicus*, *P. osmolskae*	Some species (*P.* cf. *calpensis*, see [61]: fig. 32: 3c) display the largest premolar foramen among Lagomorpha (up to few mm in diameter; Fig. 11C). In most species the foramen occurs medially to the area between P3/P4 interalveolar septum and M1 alveolus (medially to P3 in *P. tobieni*). Compared with ochotonids, *Prolagus* has the premolar foramen generally located farther distally and nearly level with the major palatine foramen, which it exceeds in size (Fig. 11C).	[58], [61], [66], [84]–[88]
*Russellagus*	**+** in *R. vonhofi*	No data on exact location	[10]
*Sinolagomys*	**+** in *S. kansuensis*, *S. ulugurensis*; **?** in *S. gracilis*, *S. major*, *S. pachygnathus*	In *S. kansuensis* the premolar foramen is mostly medial to P4 or less often medial to P3/P4 interalveolar septum. In *S. ulugurensis* the premolar foramen is medial to the P3 distal margin or to the P3/P4 interalveolar septum.	[7]: 80; [89]: fig. 1

Symbols: ‘**+**’, foramen present in all examined specimens; ‘**−**’, absent in all examined specimens, ‘**?**’ not known, ‘**∼**’ variable.

*Ochotona* and *Ochotonoides* form the core of Ochotonidae; *Ochotona* is the sole living representative of the family, and *Ochotonoides* is a closely related genus [26], [47]. In both genera the premolar foramen is well-expressed and placed medially to the P4 alveolus (see Fig. 1 for *Ochotona*). Well-preserved cranial material of *Ochotonoides* is relatively rare, but the premolar foramen was reported as present in *Ochotonoides complicidens* [48] and in “*Ochotona*” *chowmincheni* (Fig. 1B; [49]), a species similar to *Ochotonoides* in the morphology of the skull and dentition (see [26]). The presence of the premolar foramen is a very stable character in *Ochotona*. It was detected in all extant and most of the extinct species that have sufficient cranial remains available to study. The premolar foramen in *Ochotona* and in *Ochotonoides complicidens* is usually symmetric and occurs medially to the P4 alveolus, although some weak asymmetry has been detected in *O. dauurica*, *O. pallasi*, *O. pusilla*, *O. rufescens*, and *O. thibetana*, in which one of the foramina occurred at the P3 or P3/P4 level while the other was located slightly distally, at the P4 (in most cases), or at the P4/M1 level (in *O. pusilla*, AMNH 176280). A similar observation was made also by Bohlin ([7]: 80) who noted that in one specimen of *O. hyperborea mantschurica*, regarded by him as “abnormal”, the right foramen was “in normal position [at P4 alveolus], whereas the left lies even anterior to P3”. Apart from such irregularities in the position of the normally developed premolar foramina, some specimens of *O. collaris* had either very weakly developed or missing foramina (Fig. 1C), a condition not mentioned for this genus so far. In two specimens (AMNH 125717 and 125720) only a single premolar foramen exists on the left side of the palate, at P4 and P3 level, respectively. In one specimen of *O. collaris* (AMNH 137268), the premolar foramina were virtually absent on both sides, leaving only minuscule and apparently not functional traces of these structures (Fig. 1C). The skulls of all these specimens were otherwise fully developed and normal.

#### Leporidae

The premolar foramen is absent in the earliest (Oligocene) representatives of Leporidae. It was not found both in *Litolagus* [40], [43] and *Ordolagus*, which, however, has limited cranial material [50]. The phylogenetic position and content of Archaeolaginae, a paraphyletic assemblage of the leporid stem lineages [21] is unclear. Dawson [9] included in this subfamily seven genera, while Averianov [21] recognized only four of them: *Archaeolagus*, *Hypolagus*, *Lepoides*, and *Pewelagus*. He considered *Notolagus* and *Paranotolagus* the members of the Leporini tribe (he did not mention *Panolax*). We follow Averianov [21] in his treatment of the leporid phylogeny until a more comprehensive analysis is performed. The premolar foramen is absent in *Hypolagus*, *Lepoides*, and *Pewelagus* [43], [51]–[53]. Also, the condition of the premolar foramen in rare and poorly known *Panolax* is not known, as it is mainly represented by the dental material [8], [52].

*Archaeolagus*, the nominal genus of Archaeolaginae displays well-developed premolar foramina. The paired foramen is placed at the level of P3/P4 junction, more closely to the distal margin of the P3 alveolus in the holotype of *A. ennisianus* (AMNH FM 7190) from the Arikareean deposits of the John Day Formation, Oregon (Fig. 12A). The presence of these foramina is mentioned by Gawne ([12]: 14), but not so by Dawson [43] who examined the specimen and mentioned only the palatine foramina placed posteriorly to the maxillo-palatine suture ([43]: 43). Furthermore, the premolar foramen is present medially to the P3 alveolus in three specimens assigned to *Archaeolagus* cf. *macrocephalus* (AMNH F:AM 22276, 22277, 22292; Fig. 12B).

Among Leporinae, the crown group of Leporidae, the premolar foramen has not been detected in such extinct taxa as: *Nekrolagus*, *Notolagus*, and *Pratilepus* [43], [51], [54]. In *Alilepus*, a single foramen situated medially to the distal margin of P2 alveolus at the right side is present in the holotype skull of *Alilepus hibbardi* [54]. There are no data on the premolar foramen in other *Alilepus* species and the material available for us to study was very limited. Among eleven genera of modern Leporidae, the premolar foramen is variably expressed, presenting diverse incidence (0–100%) and considerable variability in morphology (Figs. 2, 3, 4). Accessory foramina are frequent. The premolar foramen is absent in all examined specimens of *Bunolagus monticularis*, *Caprolagus hispidus*, and *Romerolagus diazi*. However, only small samples of *Bunolagus* and *Caprolagus* were available for us to study. These species are extremely scanty in the zoological collections because of their critically endangered (in the case of *Bunolagus*) and endangered (*Caprolagus*) IUCN status [55]. Table 3 presents data for extant Leporidae; we comment in more details on two species-rich genera below.

**Table 3:** Presence of the premolar foramen in extant Leporidae.

Species	*N* of specimens examined/*N* of specimens with the foramen	Comments
*Brachylagus idahoensis*	25/0	Absent in all adult specimens. Minute accessory foramen present at the left side in juvenile specimen (AMNH 140865), medial to the DP3 (see Fig. 13C).
*Bunolagus monticularis*	2/0	
*Caprolagus hispidus*	2/0	
*Lepus alleni*	3/0	
*L. americanus*	20/0	
*L. arcticus*	29/0	
*L. brachyurus*	5/0	
*L. californicus*	23/0	
*L. callotis*	25/2	Symmetric at P2 or P2/P3 level, (Fig. 3A)
*L. capensis*	10/0	Absent according to Corbet [13]
*L. coreanus*	7/1	Asymmetric single and minute (L), P2/P3 level
*L. europaeus*	97/0	
*L. flavigularis*	20/4	Symmetric or asymmetric (including single) at P2/P3
*L. hainanus*	12/0	
*L. insularis*	13/13	
*L. microtis*	20/3	Asymmetric 2 single (R, L), 1 paired with (L) foramen larger, P2/P3; (Fig. 3C)
*L. nigricollis*	15/2	Asymmetric single (L, R) at P2 or P3 level; (Fig. 3D)
*L. oiostolus*	15/1	Asymmetric single (R) at P2 level, (Fig. 3B)
*L. saxatilis*	19/1	Asymmetric paired, (L) minute at P2/P3, (R) normal at P3
*L. sinensis*	5/0	
*L. timidus*	5/0	
*L. tolai*	15/0	
*L. townsendii*	9/0	
*L. yarkandensis*	n/a	Variable according to Corbet [13]; not investigated in this paper
*Nesolagus netscheri*	2/0	According to Corbet [13]; small and variably expressed. A series of small foramina medial to the left and right dental rows between P2 and P4 alveoli
*N. timminsi*	1/0	In AMNH 276142 a minute accessory foramen medial to the P2/P3 interalveolar septum at the right side, accompanied by a number of small pits at the P3 level and between the P3 and P4 alveoli
*Oryctolagus cuniculus*	26/0	Among all wild-caught specimens examined in the AMNH collection only one adult specimen (AMNH 165636) expressed poorly developed, asymmetric, minute pits (accessory foramina); two are located at the left side between P2 and P3 and medial to the P3 alveoli, while one is present at the right side of the palate medial to anterior margin of P3; absent according to Corbet [13]
*Pentalagus furnessi*	2/0	The paratype skull (ANSP 20645) has a series of small accessory foramina medial to the P4–M1 alveoli at both sides of palate; absent according to Corbet [13]
*Poelagus marjorita*	35/35	Symmetric/asymmetric (9/35); paired at the P3 or P3/P4 level; in specimen AMNH 118668 a single foramen at P3 at one side and four minute openings at the other side
*Pronolagus* sp. and *P. rupestris*	19/16	Small paired foramen at the anterior margin of P2; absent according to Corbet [13]. Anteriormost position of all extant taxa
*Romerolagus diazi*	11/0	
*Sylvilagus aquaticus*	13/11	Symmetric/asymmetric, mostly paired at the P3 or P3/P4 level (Fig. 4A); lack in juvenile specimens
*S. audubonii*	92/0	Single accessory foramen in *S. a. neomexicanus* (AMNH 131890), medial to P2/P3 (R)
*S. bachmani*	18/0	
*S. brasiliensis*	99/67	Symmetric/asymmetric between P2 and P3 alveoli, (Fig. 4C)
*S. cunicularius*	31/0	
*S. dicei*	4/0	
*S. floridanus*	202/25	Symmetric/asymmetric between P2–P4, (Fig. 4B); absent according to Corbet [13]
*S. graysoni*	8/0	
*S. nuttallii*	20/0	
*S. obscurus*	16/0	
*S. palustris*	22/22	Symmetric/asymmetric, at P2/P3 level, (Fig. 4D); present according to Corbet [13]
*S. transitionalis*	lack in 13/13	Sporadic, minute and asymmetric nutrient foramina

The premolar foramen is relatively rare in *Lepus*; Corbet [13] reported it as a variable character in *Lepus yarkandensis*. We found it present in eight more species of modern *Lepus* (Table 3; Fig. 3), discussed below. When the premolar foramen is present, it is usually variably expressed, irregularly placed, and asymmetric. This structure was not detected in *L. alleni*, L. americanus* (including *L. a. bairdi*), *L. arcticus*, L. brachyurus*, L. californicus*, *L. capensis*, *L. crawshayi*, L. europaeus*, *L. hainanus*, *L. insularis*, *L. peguensis*, *L. sinensis* (excluding *L. coreanus*), L. timidus*, *L. tolai*, and *L. townsendii*. Corbet [13] confirms the lack of this structure in five species (marked above with asterisks). It is not clear whether he regarded *L. coreanus* as a subspecies of *L. sinensis* (see [55] for the clarification of taxonomic status) when he published his observations; nonetheless, in the AMNH collection, one specimen of *L. coreanus* (cataloged as *L. sinensis coreanus*, AMNH 34109) displays a minute single premolar foramen (Table 3). The foramen is located at the left side of the transitional area between P2/P3 alveoli. The premolar foramen was also found in *L. microtis*, where it is located either unilaterally (AMNH 80834, 80836; Fig. 3C) or symmetrically (AMNH 80843), at the P2 or P2/P3 level in both cases. In *L. saxatilis* (including *L. s. megalotis*) the premolar foramen is very rare (Table 3). Only in one specimen (AMNH 168912) two foramina were found on the left side of the palate. The anterior one is slightly larger and placed lingually to the transition area between P2 and P3 alveoli. The other one is positioned more medially and at the right side of the P3 alveolus. In *L. nigricollis* (including *L. n. ruficaudatus*) the foramen is rarely present (Table 3). The specimens which actually display the premolar foramina have this character developed unilaterally as a single opening. In AMNH 102448 it appears on the left side, medially to P2 alveolus, while in AMNH 102449 it appears on the right side of the palate (Fig. 3D), medially to the distal margin of the P3 alveolus. Similarly, a rare premolar foramen was observed in *L. oiostolus*, in which it was registered only in one specimen (AMNH 113705), where it was present only at the right side of the palate, medially to the P2 alveolus (Fig. 3B). In *L. flavigularis*, the premolar foramen was present only in three (AMNH 145611, 145612, 144586) of the studied specimens (Table 3). The foramina are minute, located symmetrically medially to the P2/P3 interalveolar septum level, or unilateral placed right at the P2 (in AMNH 1456511), or paired, asymmetrically placed at both sides, left at the P2/P3 level and right at the P3 alveolus. In AMNH 145604 and 145168 there is a series of minute accessory pits between P2 and P3 (AMNH 145604) or they spread in line between P2 and M1 alveoli (AMNH 145168). *L. callotis* (including *L. mexicanus* and *L. gaillardi*), regarded as closely related to *L. flavigularis*, also displays rare premolar foramina. The foramina are placed symmetrically at the distal margins of P2 alveoli (AMNH 14148) or between the P2 and P3 alveoli (AMNH 35158; Fig. 3A).

Similar to *Lepus*, *Sylvilagus* shows a range of variability in the premolar foramen presence and development. Corbet [13] describes the condition of the premolar foramen in five species of *Sylvilagus*, whereas we were able to determine its presence in 12 out of 17 recognized species (Table 3; see [55] for taxonomic reference). The remaining five species (*S. cognatus*, *S. insonus*, *S. mansuetus*, *S. robustus*, and *S. varynaensis*) belong to small, isolated, frequently endemic, and endangered populations which were previously included either in *S. brasiliensis* or *S. floridanus* [55], and are rare in the zoological collections. Among the examined species of *Sylvilagu*s, the premolar foramen was absent in: *S. bachmani*, *S. cunicularius* (including *S. insolitus*), *S. dicei*, *S. graysoni*, *S. nuttallii*, and *S. transitionalis*. Additionally, it was absent in all but one (AMNH 131890) of specimens of *S. audubonii*, thus we regard this character as extremely rare in the species. The specimen in question, assigned to *S. audubonii neomexicanus*, has a single premolar foramen at the right side at the P2/P3 level. Among *Sylvilagus* species, the premolar foramen is present in all studied adult specimens of *S. aquaticus* and *S. palustris* (Fig. 4A, D, respectively). Corbet [13] confirms the presence of the premolar foramen in these species, and additionally reports it from *Sylvilagus brasiliensis* describing its morphology as “present and clear” ([13]: 12). We studied the presence of the foramen in *Sylvilagus brasiliensis*, including the subspecies: *S. b. andinus*, *S. b. capsalis*, *S. b. canarius*, *S. b. chillae*, *S. b. gibsoni*, *S. b. gabbi*, *S. b. meridensis*, *S. b. paraguensis*, and *S. b. truei*, and found it in 67% of the specimens (Table 3). In half of them it was asymmetric but paired (Fig. 4C); only in 8% of specimens it is single; it was located between P2 and P4 alveoli. Corbet [13] stated lack of the premolar foramen in *Sylvilagus floridanus*. However, we studied over 200 specimens of this species, including 11 subspecies (*S. f. floridanus*, *S. f. alacer*, *S. f. cumanicus*, *S. f. mallurus*, *S. f. mearnsi*, *S. f. nigronuchalis*, *S. f. similis*, *S. f. orinoci*, *S. f. orizabae*, *S. f. purgatus*, *S. f. restrictus*) and found the premolar foramen in 12% of specimens (Table 3; Fig. 4B), including seven subspecies (some of them strongly underrepresented in our sample).

### Ontogeny

Although we did not study lagomorph fetuses, and the cranial development of Lagomorpha is outside the scope of this paper, we think it advisable to offer some points on ontogeny of the premolar foramen on the basis of the osteological collection of AMNH. We studied morphology of the foramen in juvenile specimens still having deciduous dentition, most of them before weaning. Overall, incidence of the premolar foramen in juvenile specimens is similar to that in adult ones; however, we observed some exceptions. The premolar foramen was present in all specimens of juvenile *Ochotona* (Fig. 13A), *Poelagus*, and *Sylvilagus palustris* (Fig. 13B). The premolar foramen was also present in one specimen of juvenile *Sylvilagus floridanus mallurus* (AMNH 37298) but it was absent in other specimens of *S. floridanus*, which is not surprising, given that in this species the premolar foramen is present in the minority of individuals (see Table 3). On the other hand, the premolar foramen was absent in two juvenile specimens of *Sylvilagus aquaticus* (AMNH 9958 and AMNH 9937), a species in which the incidence of the premolar foramen reaches 85%. Thus, it is possible that in some cases its development may somewhat lag in ontogeny. Most species that lack the premolar foramen in adults do not have it at the juvenile stage either. The only two exceptions were a juvenile specimen of *Brachylagus idahoensis* (AMNH 140865), which has a single minute foramen at the left side of the palate (Fig. 13C), and a juvenile individual of *Oryctolagus cuniculus* reported by Wible ([15]: 228), which also expressed small foramina. Such examples are extremely rare, but they lend support to our opinion that the loss of the premolar foramen in some taxa of crown Lagomorpha was secondary.

Basically, the structure of the premolar foramen in juvenile specimens resembles that in adults, although the exact course of the premolar canal seems slightly different in juvenile *Sylvilagus palustris*. In AMNH 253183 specimen the entire canal was embedded in the delicate and spongy medial wall of the alveolar process of the maxilla, not continuing more medially to the bottom of the infraorbital groove, as it was observed in an adult specimen (Fig. 5B). Such a difference could result either from individual variability associated with overall plasticity of cranial vascularization in mammals, or the course of the canal will shift to the floor of the infraorbital groove during development.

## Discussion

### Function

When it is present, the premolar foramen is usually very small, not exceeding 0.5 mm in diameter, and thus could not form the passage for any major blood vessel, especially taking into consideration the dense, branching, and rather plastic pattern of cranial vasculature [56]. Only in *Prolagus* the enlarged premolar foramina reach a considerable diameter, even exceeding well-developed palatine foramina in this genus, similar to the largest palatine foramina in some leporids. Thus, it is safe to assume that in these species they could be of more importance for the cranial circulation.

The actual function of the premolar foramen has been poorly researched. Bohlin ([7]: 60) implied that the function of the premolar foramen in ochotonids must be of certain importance, because he observed that it is universally present in all available specimens. However, in this paper we report numerous specimens which do not have the premolar foramen, but belong to the species in which that structure is reported. Moreover, we found some representatives of the species (e.g., *Ochotona collaris*) that usually have the premolar foramen, in which the foramen either lacks totally or is a nearly obliterated, non-functional structure (Fig. 1C). Thus, it can be assumed that the function of the premolar foramen is not as crucial as it was previously thought, at least in species in which the foramen has relatively small diameter.

**Figure 14:**
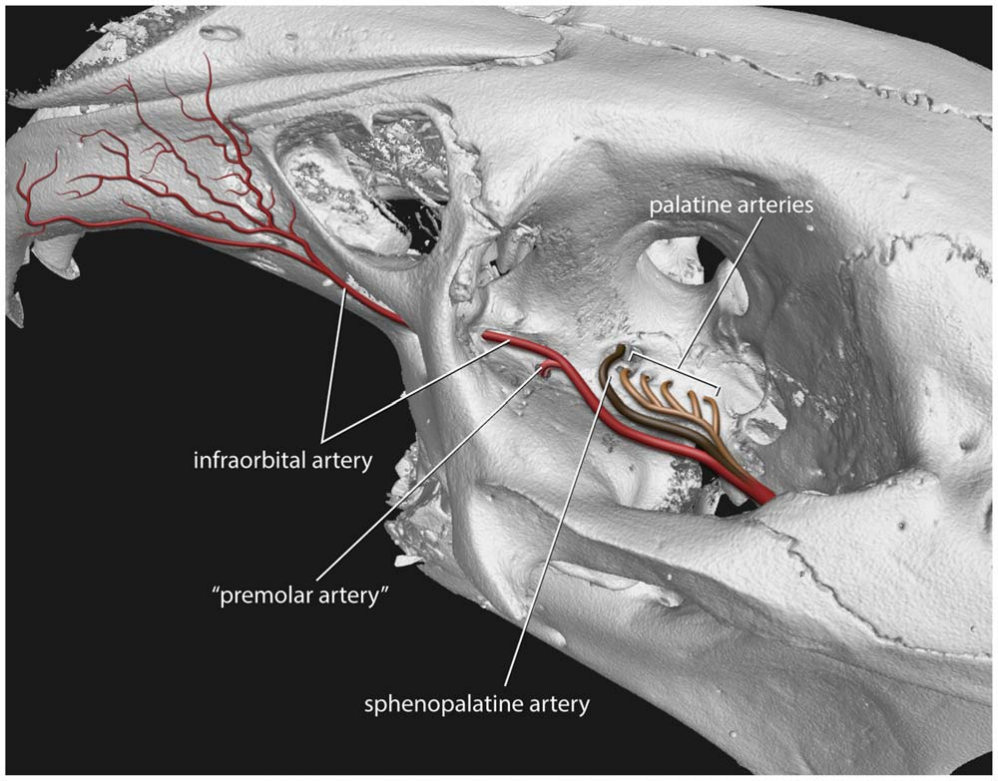
Scheme of the maxillary artery branching in the infraorbital groove of *Ochotona*. Based on μCT reconstruction of the *O. princeps* (AMNH 95203).

The infraorbital groove is supplied by the maxillary artery which upon entering the groove splits into two main branches. The first branch, the infraorbital artery, goes laterally and slightly dorsally and passes the alveolar process of the maxilla medially. The second branch is the common trunk of the major palatine artery and the sphenopalatine artery, which branches off medially and ventrally (Fig. 14).

The micro-CT scanning study of *Ochotona* and *Poelagus* skulls points to the origin of the ‘premolar artery’ as an offshoot of the infraorbital artery (Figs. 7, 14). The dorsal aperture of the premolar canal in both studied genera lies more anteriorly than the dorsal aperture of the canal hosting major palatine arteries, the pterygopalatine foramen in *Poelagus* (Fig. 2) or multiple small foramina in the case of *Ochotona* (Fig. 14), or the sphenopalatine foramen. In *Poelagus* the pterygopalatine foramen lies dorsally to the M1/M2 interalveolar septum, the distal edge of the sphenopalatine foramen lies dorsally to the distal part of M1, whereas the dorsal aperture of the premolar foramen is located dorsomedially to the P4/M1 interalveolar septum. In *Ochotona* the sphenopalatine foramen and the anteriormost dorsal aperture of the canal hosting the descending palatine artery lies dorsomedially to the P4/M1, whereas the dorsal aperture of the premolar foramen is located more laterally and anterodorsally to the P3/P4 interalveolar septum. In both genera the dorsal aperture of the premolar foramen lies anteriorly and laterally to the splitting point of the major trunk of sphenopalatine and major palatine arteries from the infraorbital artery, exactly on the course of the latter (Fig. 14). The infraorbital groove, extremely constrained medio-laterally in *Poelagus*, clearly shows the infraorbital canal that briefly merges with the premolar canal, the latter hosting an offshoot of the infraorbital artery which descends in the antero-ventral direction and passes through the premolar foramen at the interalveolar septum of P4/M1 (Fig. 7F).

Cranial circulation in the palate and infraorbital groove has not been studied in *Ochotona* or *Poelagus*, but it is well-known in the domesticated rabbit (*Oryctolagus cuniculus*) which does not display the premolar canal [56], [57]. In *Oryctolagus* the only larger artery that arises from the infraorbital artery in the region between P3 and M1 is the anterior superior alveolar artery [56], [57]. This artery leaves the orbit through the anterior alveolar canal which exits much more anteriorly, in front of the tooth row, but its general course makes it (or one of its secondary branches) a probable candidate for the ‘premolar artery’. Further, the premolar foramina appearing in the vicinity of P2, as in *Pronolagus*, may support this hypothesis.

In terms of function, we can assume that the premolar foramen plays rather a secondary role in the supplementation of the palatal region, which explains high variability of this character in some genera (e.g., in *Lepus* or *Sylvilagus*). Furthermore, the presence of the premolar foramen is not directly related to the length and structure of the hard palate, it is also not directly related to the number, size and placement of major and minor palatine foramina. In extant *Ochotona*, palatine foramina are numerous and very small, and located much more posteriorly than the premolar foramen, thus it can be assumed that in this particular lineage premolar foramen might have hosted the artery supplementing the hard palate in a more efficient way.

On the other hand, the recent leporids show interplay between the presence of the premolar foramen and the condition of palatine foramina. In *Poelagus*, which has the premolar foramen, the palatine foramina are relatively small, but still functional, thus premolar foramen might have played the supporting role in blood supplementation of the discussed area. In *Romerolagus*, which lacks the premolar foramen, the palatine foramina are relatively large. Nevertheless, among *Lepus* and *Sylvilagus* species the situation is mosaic and inconclusive, as most species usually have well developed palatine foramina with a major pair placed at the maxillo-palatine suture and their structure and size is not affected by presence or absence of premolar foramina (single or paired; Figs. 3, 4).

The premolar foramen might have played a more important role in vascularization of the oral cavity in certain species of *Prolagus* (e.g, *P. calpensis*, *P. ibericus*, *P. imperialis*, and *P. sardus*) as evidenced by its significant enlargement. In these species, the increase in size of the premolar foramina is paralleled by still large palatine foramina (Fig. 11C), thus we can assume that the vascularization of the oral cavity (specifically, soft tissues of the palate) was additionally enhanced. This situation can be interpreted in terms of improved means of thermoregulation in species living in more arid and warmer conditions then their forest-dwelling ancestors [58]. The animals could more effectively cool themselves by panting, due to increased blood flow through the mucous membranes in the oral cavity. It might have been an important adaptation in rather hot Mediterranean climate at that time.

### Phylogenetic implications

**Figure 15:**
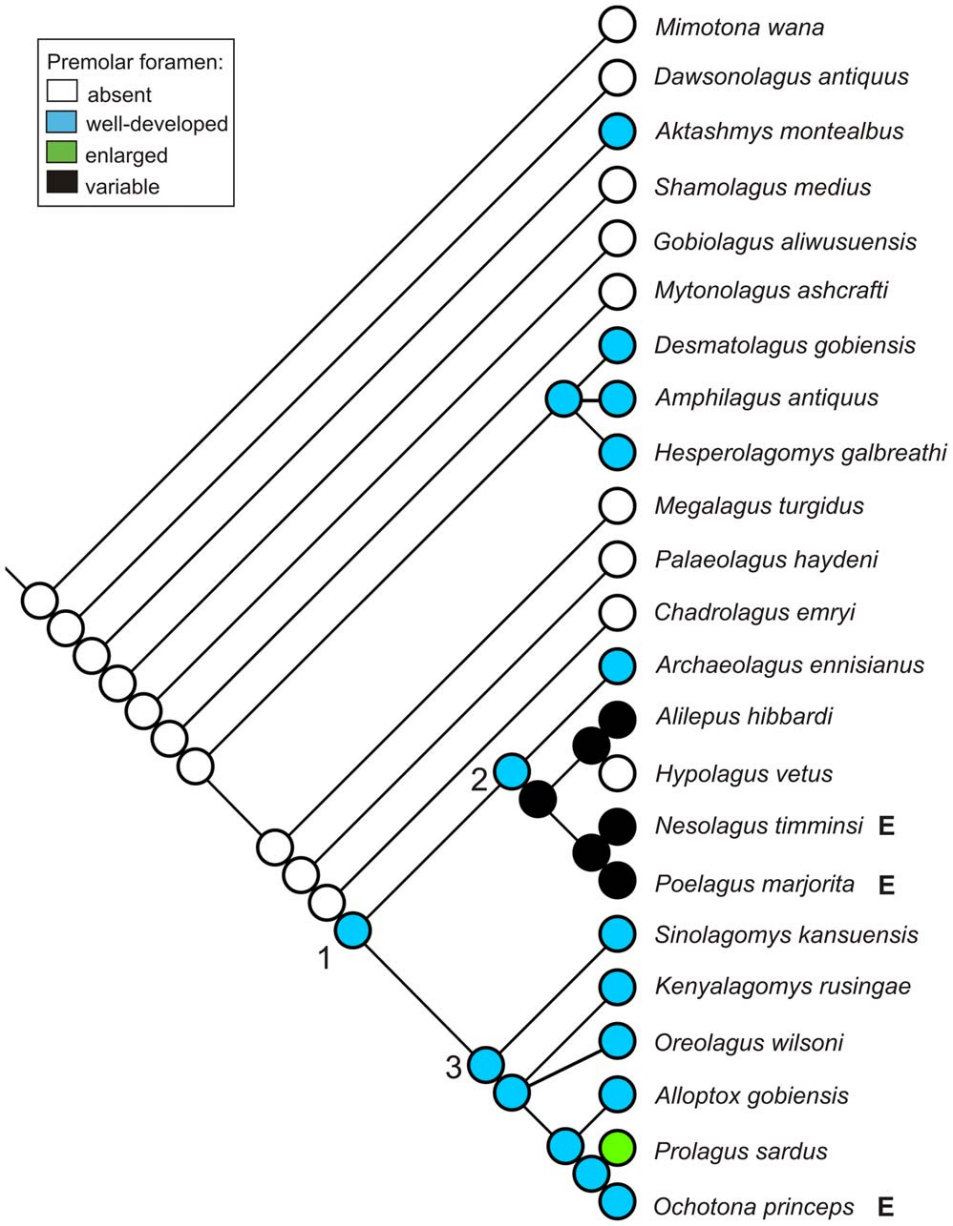
Evolution of presence and size of the premolar foramen in fossil Lagomorpha. Character ancestral states were reconstructed using parsimony and mapped on strict consensus tree of 172 equally parsimonious trees; tree length = 284, consistency index = 0.57, retention index = 0.73, resulting from parsimony analysis of 80 morphological characters for 23 taxa (1 outgroup taxon); branch lengths are arbitrary; heuristic search using subtree pruning and regrafting (SPR) algorithm; all characters unordered and equally weighted. Three main groups are numbered: 1 – crown Lagomorpha, 2 – Leporidae, 3 – Ochotonidae. E, extant.

**Figure 16:**
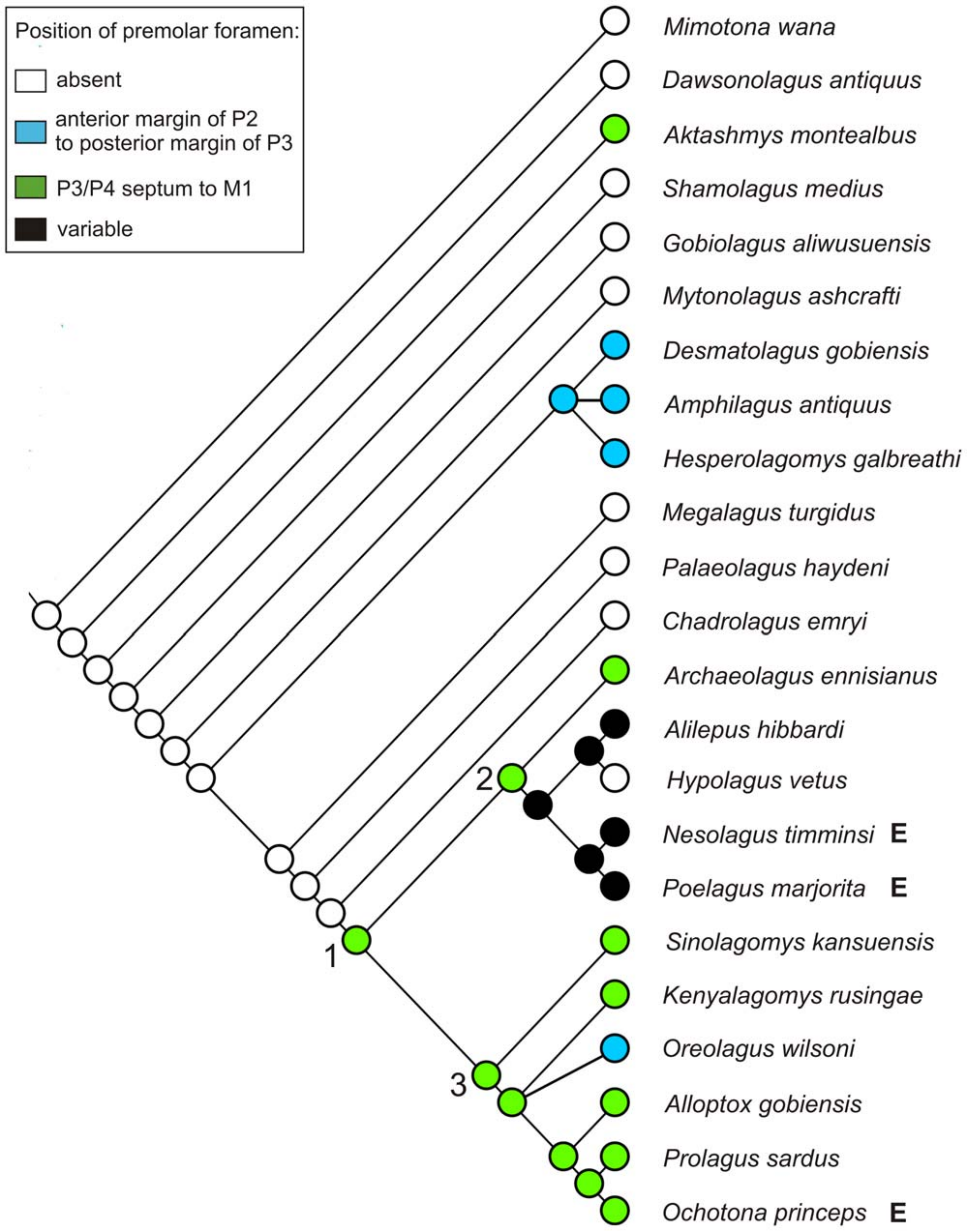
Change of position of the premolar foramen in fossil Lagomorpha. Parameters and explanations as in Fig. 15.

Our analysis (Figs. 15, 16) does not support the premolar foramen as a synapomorphy for ochotonids. Instead, we suggest rejecting it as such a feature on the basis of its wide distribution across Lagomorpha as we present herein. The premolar foramen is indeed observed in all the genera currently included in Ochotonidae and Prolagidae ([55], see [1] for fossil representatives of the former family), but it is also present in many extant (Table 3) and fossil Leporidae (Figs. 2, 3, 4, 5A–B, 7, 8, 12). Moreover, the premolar foramen was found among early representatives of stem groups, such as *Aktashmys montealbus* [5], *Gobiolagus lii* [59], and disputable specimen IVPP V 8430 of *Gobiolagus tolmachovi* [16], [60], and in several Oligocene–Miocene genera, such as *Amphilagus*, *Desmatolagus*, *Eurolagus*, and *Titanomys* (Table 1; Figs. 9, 10), customarily regarded as Ochotonidae, partly on the basis of presence of the premolar foramen [9], [49], [61]. However, on the basis of the dental and cranial characters and our analysis (see Tree S1), we regard all the aforementioned taxa as belonging to stem lagomorphs (Figs. 15, 16; see also [62], [63]).

We note that there is still no comprehensive lagomorph phylogeny including fossil taxa and based on extensive morphological evidence. A few extinct taxa (mainly *Palaeolagus*, *Prolagus*, and *Sinolagomys*) have been included into large-scale Glires phylogeny [2], [24], [37]. In order to map the incidence of the premolar foramen onto the main lineages of the crown and stem groups, we performed a preliminary cladistics analysis (see Tree S1; Figs. 15, 16). The results obtained by us show that certain taxa regarded so far as the primitive ochotonids (e.g., *Amphilagus* and *Desmatolagus*) are outside the crown group (see Figs. 15, 16), regardless their premolar foramen condition.

The observations conducted across taxonomic diversity of Lagomorpha made it clear that the premolar foramen is not a unique and invariable character in ochotonids and it is probably better to treat it as a plesiomorphic character for this clade. The ancestral state of the premolar foramen in Lagomorpha, according to our analysis, is its absence (Fig. 15). In the older Eocene taxa, in which the premolar foramen can be recognized, it was located in the palate region between P3 and P4 alveolus. The anterior shift of the premolar foramen to the region of the P2 alveolus occurred relatively early in the history of Lagomorpha, in the Oligocene *Amphilagus* (Fig. 10A). It is also observed in some lineages of the crown leporids (*Pronolagus*). The posterior shift in the P4 and P4/M1 vicinity, which is now characteristic of Ochotonidae, was probably ancestral state for all the crown Lagomorpha (Fig. 16).

**Figure 17:**
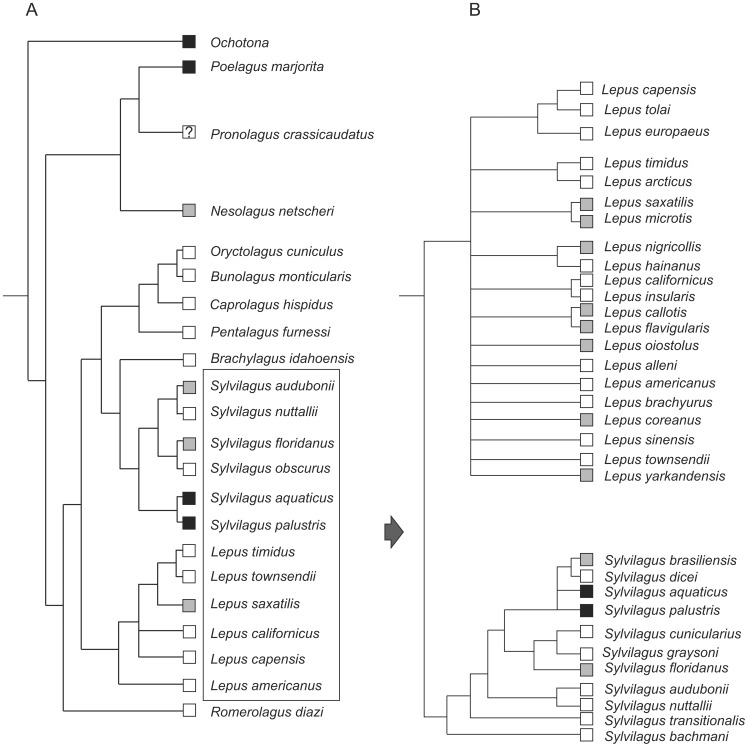
Premolar foramen distribution in extant Lagomorpha. A. All living genera, supertree from [25], modified. B. *Lepus* and *Sylvilagus*, tree from [23]. Black, the foramen present in all examined specimens; white, absent in all examined specimens; gray indicates presence in some specimens; ? denotes uncertainty about homology of the opening.

We could hypothesize that the presence of the premolar foramen characterizes the “more advanced” lagomorph stem taxa, including *Amphilagus*, and *Desmatolagus* lineages, their common ancestor and the crown Lagomorpha, in which its appearance is mosaic due to the secondary loss of the premolar foramen in some leporid lineages (Figs. 15, 16). However, the presence of that character in some Eocene taxa such as *Aktashmys montealbus* and *Gobiolagus lii* suggests a repeated origin of the feature within Lagomorpha. Moreover, as demonstrated by extant taxa, the premolar foramen is a highly variable character that could have been lost in some groups (e.g. *Hypolagus*; Fig. 15), a situation we observed in some specimens of modern species. That said, in some cases the premolar foramen does have phylogenetic meaning when its morphology and position in closely related taxa (in sister species, e.g., *Sylvilagus palustris* and *S. aquaticus*; Fig. 17) are considered.

The posterior shift of the premolar foramen within the palatine process of the maxilla was observed in a couple of lineages, such as *Desmatolagus* [64], *Oreolagus* [65], and *Prolagus* [66]. The observed posterior shifts were mainly by one tooth locus and occurred in the geologically younger species. Huang [64] noted that the premolar foramen in *Desmatolagus gobiensis* occurs mainly at the P3 alveolus while in *D. pusillus*, the stratigraphically younger species, it appears mainly at the P4 alveolus. The similar shift was observed in North American *Oreolagus*, where Hemingfordian species (*O. wilsoni* and *O. nebrascensis*) displayed the premolar foramen positioned medially to the anteroloph of P3, while Barstowian species (*O. nevadensis* and *O. wallacei*) displayed foramina at the posteroloph of P3 or at the P3/P4 alveolar septum respectively [65]. The posterior-most premolar foramen occurs in *O. wallacei* which is the youngest species, taking into account its fossil record [65], thus so far the evolutionary trend is confirmed for this lineage. The similar observations were made for *Prolagus* in which also the enlargement of the premolar foramina was observed ([66]; Fig. 11A–B). The primitive Miocene members of the genus (such as *P. oeningensis*) have a relatively small premolar foramen medial to P3/P4 alveolar septum (Fig. 11A), not different in size from that in ochotonids, but the Pliocene and Pleistocene taxa (e.g., *P. ibericus* or *P. sardus*) have considerably enlarged, oval-shaped foramen, located at the P4 (Fig. 11C). We agree with Angelone and Sesé [66] who argue that the morphology of the premolar foramen in *Prolagus* can be of some taxonomic importance, especially while speaking of coeval and co-occurring species. Nevertheless, in all these cases, the posterior shift of the premolar foramen within the palatine process of the maxilla is the secondary effect due to the posterior extension of the incisive foramen. The evolutionary trend towards reduction of the hard palate expanse is one of the noticeable regularities observed in the skull of Lagomorpha [40], [43]. It can be perceived as interplay between increase of the posterior reach of the incisive foramina and the anterior extension of the choanae, and is differently attained in particular lineages. Although the shortened hard palate is a condition characteristic for lagomorph crown group as a whole, the exact position of the hard palate and thus the posterior reach of the incisive foramen and the anterior margin of choanae is highly variable among the crown taxa. Overall, in leporids the distal margin of the choanae shifts anteriorly, while the extent of the incisive foramen remains stable, whereas in ochotonids the incisive foramen expands posteriorly. The position of the premolar foramen is derivative of the structure of the hard palate, in particular the maxillary component. In *Prolagus* and some ochotonid lineages the incisive foramen increases visibly backward even to the P3/P4 or P4/M1 (Figs. 1A, B, 11), considerably restricting the space for the premolar foramen to occur and thus forcing it posteriorly. Surprisingly, this tendency does not go in parallel with the stratigraphic record in the *Ochotona* lineage. The situation seems to be mosaic in this group, reflecting probably more complex phylogenetic history or the secondary decrease of the posterior reach of the incisive foramen in certain lineages (e.g., *O. collaris*; Fig. 1C). *Ochotona nihewanica*, one of the early species of *Ochotona* from the Miocene of China, regarded also as one of basal species in this clade [26], has a very extended and large incisive foramen, reaching P4/M1 level (Fig. 1B), while *O. collaris*, considered as one of the most derived species [67] has the posterior margin of the incisive foramen placed relatively anteriorly, at the P3/P4 level, farther ahead than in *O. princeps* (positioned at the distal margin of P4; Fig. 1A).

Unlike ochotonids, Leporidae, in which generally the hard palate stretches between P2 and P4/M1, show greater variability in location of the premolar foramen; it can occur anywhere between P2 and M1 alveolus (Figs. 2, 3, 4, 12). However, the position along the anterior premolar loci is more frequent. Furthermore, some regularity is observed in particular lineages, e.g., in *Pronolagus*, in which a minute foramen, if present, occurs always at the anterior margin of the P2 alveolus.

To determine if the premolar foramen is homologous in Lagomorpha, we considered three alternative scenarios. The first possibility is that the premolar foramen is a homologous character in all Lagomorpha. According to the second hypothesis, the premolar foramen of all crown lagomorphs is a neomorphism (in that case the foramen would not be homologous to that of stem groups), and the third hypothesis is that the foramen is a neomorphic structure only in Ochotonidae. The principal criteria to postulate homology of this opening are its incidence, size and topology (positional relationship with the upper tooth row). We used unordered parsimony reconstruction to trace the presence, size and position of the premolar foramen in all major groups of lagomorphs (Figs. 15, 16). In our opinion, the results support homology of the premolar foramen in all crown Lagomorpha (the second hypothesis). It is possible (although not very probable) that this structure is homologous in all Lagomorpha; this uncertainty is due to insufficient resolution of the tree below the crown group. In particular, our analysis did not include some stem taxa in which the foramen is present (e.g., *Eurolagus fontannesi*, *Gobiolagus lii*, and *Titanomys*), which may have affected the parsimony reconstruction.

## Supporting Information

List S1 **Characters used in the phylogenetic analysis.** Some characters modified from [2], [15], [26], [39].
(PDF)

Dataset S1 **Character-taxon matrix of fossil and extant Lagomorpha used in the phylogenetic analysis.**
(PDF)

Tree S1 **The strict consensus of 4 equally most parsimonious trees (MPT).** Tree length (TL) = 280, consistency index (CI) = 0.5714, retention index (RI) = 0.7345. 22 lagomorph taxa are included; *Mimotona wana*, a duplicidentate Glires representative is an outgroup. The data matrix was subjected to heuristic parsimony searches with TBR branch swapping algorithm and at least 5 000 random addition replicates in PAUP* version 4.0b10 [28]. All characters unordered and unweighted and the delayed transformation (DELTRAN) optimization was used.
(PDF)
