# Supplementary material for: Comparative Morphology of Premolar Foramen in Lagomorphs (Mammalia: Glires) and Its Functional and Phylogenetic Implications
Source: PLoS One. 2013 Nov 21;8(11):e79794. doi: 10.1371/journal.pone.0079794 (PMC3836788; doi:10.1371/journal.pone.0079794)
Supplement: List S1 — Characters used in the phylogenetic analysis. Some characters modified from [2], [15], [26], [39]. (PDF) [file pone.0079794.s001.pdf]

Dentition

(1) Number of upper incisors: (0) two pairs; (1) one pair.

(2) Number of lower incisors: (0) two pairs; (1) one pair.

(3) Second pair of upper incisors: (0) relatively large; (1) significantly reduced, peg-like.

(4) P2: (0) strongly reduced, not exceeding 50% of the P3 width; (1) reduced, but up to 60% of the P3 width; (2) smaller than P3 but retaining at least 70% of the P3 width.

(5) P2 root number: (0) two roots; (1) one root.

(6) P2 crown morphology: (0) cuspal morphology; (1) persistently trilobate; (2) bilobate in adults, trilobate in juveniles and subadults; (3) bilobate.

(7) Lingual lobe of P2: (0) absent; (1) rounded, oval, or semicircular, in adults similar in morphology and size to the central lobe; (2) enlarged and semicircular without any deep secondary folds; (3) enlarged oval or rectangular, secondary folds of variable depth.

(8) P2 occlusal surface: (0) not compressed, heart-shaped or round; (1) antero-posteriorly compressed; (2) variable.

(9) P3 molarization: (0) nonmolarized, lingual lobe less than 50% of the tooth width toward buccal wall, crescentic valley open; (1) nonmolarized, lingual lobe reaches over 50% of the tooth width, crescentic valley closes late in ontogeny or remains open; (2) semimolarized, molarization delayed in ontogeny until subadult stages; (3) fully molarized.

(10) P3 hypostria morphology: (0) lack of hypostria, lingual margins convex; (1) nascent, expressed as a wide concavity; (2) hypostria shallow but defined, not forming lakes; (3) hypostria shallow, quickly forming lakes; (4) deep (not less than one-third of the width of the occlusal surface), persistent, and not forming lakes.

(11) Additional fold on the anterolingual margin of the P3 occlusal surface: (0) present; (1) absent.

(12) Crescent on P3: (0) absent; (1) more persistent than hypostria; (2) less persistent than hypostria, quickly vanishing in ontogeny; (3) equally persistent as hypostria.

(13) P3 root morphology: (0) two buccal rootlets located symmetrically on the shaft; (1) two buccal rootlets located asymmetrically on the shaft; (2) one buccal rootlet, or extended shared base giving rise to two rootlets; (3) buccal rootlets lost.

(14) P4 molarization: (0) nonmolarized; (1) semimolarized; (2) fully molarized.

(15) P4 root morphology: (0) two buccal rootlets; (1) one buccal rootlet or extended shared base giving rise to two rootlets; (2) buccal rootlets lost.

(16) P4 hypostria morphology: (0) hypostria absent; (1) shallow, weakly marked; (2) defined, but quickly closing and forming a lake; (3) relatively deep (not exceeding 50% of the crown width), no lakes; (4) very deep (cutting over 70% of the crown width).

(17) M1 and M2, morphology of hypostriae: (0) shallow, up to 25% of the crown width; (1) deeper but quickly closing, forming lakes; (2) deep and open throughout ontogeny.

(18) P4–M2 hypostriae versus crescent: (0) lack of crescent; (1) crescent more persistent than hypostria or hypostrial lakes; (2) hypostria and/or hypostrial lake more persistent than crescent.

(19) Hypostriae development: (0) no hypostriae; (1) hypostriae display unequal depth on different loci during wear; (2) depth of the hypostriae is similar on all loci throughout ontogeny.

(20) M1 buccal roots: (0) two sturdy roots, close to the occlusal surface; (1) two delicate roots, attached to the shaft most of their length; (2) absent.

(21) M2 buccal roots: (0) two roots detaching from the shaft at the same level, close to the occlusal surface; (1) one asymmetrical root or two small roots located asymmetrically at the shaft; (2) no roots.

(22) M2 morphology: (0) similar to M1, slightly smaller and more asymmetric (posteroloph narrower); (1) simplified, but bilobate, crescent lost, hypostria relatively shallow, tooth gently arching posteriorly; (2) with a third smaller lobe, pointing posteriorly.

(23) M3 size: (0) large, smaller than M2 but not more than by 15%; (1) reduced in size compared to M2 (less than 50% of M2 width), but remnants of the posteroloph and hypostria still present; (2) reduced to a single enamel column, no occlusal structures preserved; (3) absent.

(24) M3 root: (0) two buccal roots; (1) one buccal root; (2) no buccal root but keel along the shaft; (3) no roots, shaft smooth.

(25) p3 size: (0) reduced in comparison to p4; (1) not reduced.

(26) p3 occlusal surface: (0) irregular or triangular, trigonid and talonid poorly separated; (1) bilobate, trigonid and talonid well defined (hypoflexid deep, no mesoflexid); (2) bilobate, with smooth trigonid and talonid distinctly separated by deep reentrants (hypoflexid and mesoflexid), especially at earlier wear stages; (3) originally bilobate, but complex, reentrants deep, numerous (additionally protoflexid, anteroflexid, and paraflexid may appear), and persistent.

(27) p3 trigonid size: (0) distinctly smaller than talonid; (1) slightly smaller or equal to talonid; (2) larger than talonid.

(28) p3 trigonid shape: (0) rounded or irregular; (1) complicated irregular, branching with deep flexids; (2) trapezoid with flexids of variable depth, frequently crenulated; (3) triangular with simple flexids.

(29) p3 anteroconid: (0) absent; (1) rounded poorly isolated; (2) clearly isolated by deep para- and protoflexids; (3) fully separate from the trigonid.

(30) p4 root morphology: (0) two roots; (1) one root, one pulp chamber; (2) one root, two separate pulp chambers.

(31) m1 root: (0) two roots; (1) one root, one pulp chamber; (2) one root, two separate pulp chambers.

(32) m2 morphology: (0) bilobate, similar to m1, (1) trilobate.

(33) m2 root: (0) two roots; (1) one root, one pulp chamber; (2) one root, two separate pulp chambers.

(34) m3: (0) larger than m2; (1) nearly equal to m2 and of similar structure; (2) smaller than m2 and of similar structure (trigonid and talonid quickly merge at the lingual side); (3) significantly smaller than m2 and simplified (two conids, but their occlusal surfaces do not merge); (4) reduced to a single column, peg-like; (5) absent.

(35) m3 hypoconulid: (0) large and persistent; (1) small and quickly vanishing; (2) absent.

(36) Largest lower tooth: (0) m3; (1) m2; (2) p4–m2 of similar size.

(37) Lingual dental bridges: (0) absent; (1) markedly delayed in ontogeny; (2) formed early in ontogeny.

(38) Relative length of trigonid to talonid: (0) trigonid compressed antero-posteriorly, (1) both of similar length.

Skull

(39) Basicranial angle: (0) skull flat; (1) skull arched.

(40) Nasals shape: (0) narrow posteriorly, expanded anteriorly; (1) narrow with parallel sides; (2) broad posteriorly with V-shaped frontal articulation.

(41) Supraorbital process: (0) incipient; (1) relatively small, more flattened, triangular; (2) larger, extended posteriorly, frequently transformed into more complex postorbital process; (3) absent.

(42) Supraorbital notch: (0) absent; (1) present.

(43) Nuchal crest (external occipital protuberance): (0) posterodorsal, forming horizontal ridge between the parietal and occipital; (1) posteroventral, U-shaped and contained within the occipital.

(44) Maxillary fenestra pars facialis: (0) absent; (1) covered by delicate bone lacework; (2) single oval dorsal opening with or without smaller lacework part placed ventrally.

(45) Maxillary fenestra pars orbitalis: (0) absent; (1) present.

(46) Anterior maxilla (rostral part): (0) sharp wedge cutting anteriorly into the premaxilla; (1) the maxilla-premaxilla contact almost vertical or semicircular; (2) the premaxilla wedges into the maxilla.

(47) Anteroventral orbital rim: (0) round; (1) square.

(48) Lateral exposure of the petrosal: (0) large triangular in lateral aspect, oriented posterodorsally to the external meatus; (1) large semilunar in lateral aspect surrounding the external acoustic meatus posterodorsally; (2) small triangular, wedged between the parietal and the auditory bulla.

(49) Exoccipital: (0) broad, semicircular, with blunt and short paracondylar processes of the exoccipital; (1) thin, L-shaped, with long and narrow paracondylar processes; (2) relatively narrow, semicircular with elongated and pointy paracondylar processes.

(50) Paracondylar process of the exoccipital: (0) extends halfway down the auditory bulla; (1) extends all the way down the auditory bulla.

(51) Incisive foramen: (0) short, located centrally within the diastema; (1) elongated, oval or tear-shaped, extending along most of the diastema length; (2) pear-shaped, expanded posteriorly with an anterior part substantially narrower and/or separated by the medial shelves of the premaxilla.

(52) Posterior margin of the incisive foramen: (0) anterior to the P2 alveolus; (1) at the P2 alveolus; (2) immediately posterior to the P2 alveolus; (3) at the P3 alveolus or farther back.

(53) Distal part of the upper dental row: (0) straight course of the dental row, (1) strongly bends posterolingually in the molar part, (2) gently bends posterolingually, the tooth row evenly arched.

(54) Choanae position: (0) at the level of M2; (1) between M1 and M2, (2) farther anteriorly (at M1 or P4/M1).

(55) Root exposure in the orbit floor: (0) absent, (1) present.

(56) Jugal: (0) wide and massive; (1) delicate and relatively narrow.

(57) Posterior process of the jugal: (0) absent; (1) short and blunt; (2) long and sharp; (3) intermediate.

(58) Masseteric spine: (0) poorly expressed, narrow and semilunar in ventral view; (1) large and semilunar; (2) small tubercular but well-defined.

(59) Zygomatic fossa: (0) absent; (1) very shallow and poorly expressed; (2) shallow but well-marked, deeper anteriorly; (3) large and deep, covering half of the jugal.

(60) Posterior margin of the zygomatic process of the maxilla: (0) at M1 or M2; (1) at P4 or between P4 and M1.

(61) Relative length of the hard palate: (0) long, palatal bridge not less than 70% of the upper tooth row length; (1) moderate, palatal bridge equals 50–69% of the upper tooth row length; (2) short, less than 50% of the upper tooth row length.

(62) Palatine portion of the palate: (0) large, longer than the maxillary portion; (1) shortened, forming 40–55% of the palatal bridge; (2) strongly shortened, forming less than 40% of the palatal bridge; (3) entirely dominating the hard palate.

(63) Position of the palatine suture: (0) at P3 or between P3 and P4; (1) at P4 or between P4 and M1.

(64) Palatine foramina: (0) one large foramen within the palatine at each side; (1) one major and one or two smaller foramina within the palatine at each side; (2) major pair of foramina at

the maxillo-palatine suture, additional pair (if present) within the palatine; (3) multiple small foramina within the palatine.

(65) External acoustic meatus: (0) no bony tube; (1) short bony tube; (2) elongated tube directed posterolaterally.

(66) Size of the auditory bulla: (0) small, less than 99% of the upper tooth row length; (1) moderate, 100–119% of the upper tooth row length; (2) large, at least 120% of the upper tooth row length.

(67) Premolar foramen: (0) absent; (1) well-developed; (2) enlarged; (3) variable: asymmetrically placed on both sides, single or multiple, uneven size.

(68) Premolar foramen position: (0) absent; (1) between anterior margin of P2 alveolus and the posterior margin of P3 alveolus; (2) between P3/P4 alveolar septum and the M1 alveolus; (3) variable (including asymmetric).

(69) Basioccipital: (0) elongated broadest posteriorly; (1) rectangular slightly hourglass-shaped or with parallel lateral margins; (2) almost square broadest anteriorly.

(70) Lateral margin of the basioccipital: (0) flat or slightly downturned; (1) with the prominent tympanic process overlapping medial wall of the auditory bulla; (2) with thick elongated prominence meeting the downturned lip of the medial wall of the auditory bulla.

Mandible

(71) Anterior mental foramen: (0) in the diastemal part; (1) absent or expressed as a field of small pits under premolars; (2) under p3 or p3/p4 septum.

(72) Posterior mental foramen: (0) under p3 or p4; (1) under m1 or m2; (2) behind the tooth row; (3) absent, or a field of small pits below p3–m1 area.

(73) di2 ending: (0) under m2 or farther back; (1) under m1 or p4; (2) at the p3/p4 level; (3) restricted to the diastemal part.

(74) Anterior edge of the masseteric fossa: (0) under m2; (1) at the m2/m3 level; (2) at the m3 level; (3) shifted posteriorly to the ventral part of the angular process.

(75) Masseteric tubercle: (0) present as a small knob; (1) absent.

(76) Coronoid process: (0) large, blade-like; (1) moderately sized, plate-like and triangular, placed laterally to the groove for the attachment of the temporalis muscle; (2) small, tubercle-like, placed in the mid-height of the ascending ramus.

(77) Coronoid process base: (0) well posterior to m3; (1) slightly anterolateral to m3; (2) immediately posterior to m3; (3) the base poorly defined.

(78) Angular process: (0) elongated with pointy distal end; (1) large with rounded distal margin.

(79) Coronoid canal: (0) absent; (1) present.

(80) Pterygoid fovea: (0) extended, covering most of the ascending ramus; (1) restricted to the condylar process, elongated, slightly tear-shaped.
