# Supplementary material for: Comparative Morphology of Premolar Foramen in Lagomorphs (Mammalia: Glires) and Its Functional and Phylogenetic Implications
Source: PLoS One. 2013 Nov 21;8(11):e79794. doi: 10.1371/journal.pone.0079794 (PMC3836788; doi:10.1371/journal.pone.0079794)
Supplement: Dataset S1 — Character-taxon matrix of fossil and extant Lagomorpha used in the phylogenetic analysis. (PDF) [file pone.0079794.s002.pdf]

M. wana	0000?00000?0?000000??00000000??0?0000102?0?0?10???002?00?000??????00??00021???0?
A. montealbus	0??1????0011201101100001?????000010100??????????????????0?0001???12???????????
D. antiquus	01?01???0011201101100000?????0000101001?10??1?0????01000?0200001??00????02100?0?
S. medius	01?1?1100011?0?10110001?00101000020110??????????????20?????00000??00??0002001???
G. aliwusuensis	01?1111000112011011000011010101012111 0?????????????0110??1?00000??00??00010?1???
D. gobiensis	01101110010112011110001100101000031210?????????????321???0?1001???11??01010?1???
M. ashcrafti	0111111001112111111000120210000002111 0?????1???????12101?1210001??00??00010?1?0?
M. turgidus	0111111112111202111010120110000002121012?0011?10?1122001?120000?10001200120?1?0?
P. haydeni	01111210231112021211101312100110121211021001101010122101112100011100110011 01100?
C. emryi	011113102212322322122013121002202212210210?1?01???112101112100 01??00??00120?1?0?
A. antiquus	0111111101110101011 0003?1110010005?201?????????????20101?0?11001??11???????????
P. sardus	01111122121332232222203?132132212522011030011100002302002230210111221101 23123010
S. kansuensis	011?1???0213322322222 03?00000220242201??0??????????3010??0?12?????12??0012??1?0?
K. rusingae	0111131102133224222220 3?00000220242201013002 1101?0230200313021121112??2103112011
H. galbreathi	01101???020111032210103?000000000 42200??????????????2????0?1??????11??01131?1?1?
A. gobiensis	01111331021332242222203?1011022024220101000211120013020022302111021211010 31230?1
O. wilsoni	01111???02133224222221 3?00000220242201?????????????3020??2?12300??11??01?31?1???
O. princeps	0111132112133224222222 3?10232220242201003002110200230100223123130212011213123010
A. ennisianus	01121331321232232222202311221220 23?221121??10?1???1222011121121220 12??0312112???
A. hibbardi	0111132134123224222220231322122023222112201 1?111??1222111120221220332203221121??
H. vetus	01121321341232242222202313231220232221122011?011??1222?1112022102000 22032?112111
N. timminsi	011213313412322422222023131212202322211220111211211022111121120020332203311 12111
P. marjorita	0112133134123224222220231322122023222112201102112112222111120121220332203311 12111
