# Supplementary figures and images for: Comparative Morphology of Premolar Foramen in Lagomorphs (Mammalia: Glires) and Its Functional and Phylogenetic Implications
Source: PLoS One. 2013 Nov 21;8(11):e79794. doi: 10.1371/journal.pone.0079794 (PMC3836788; doi:10.1371/journal.pone.0079794)

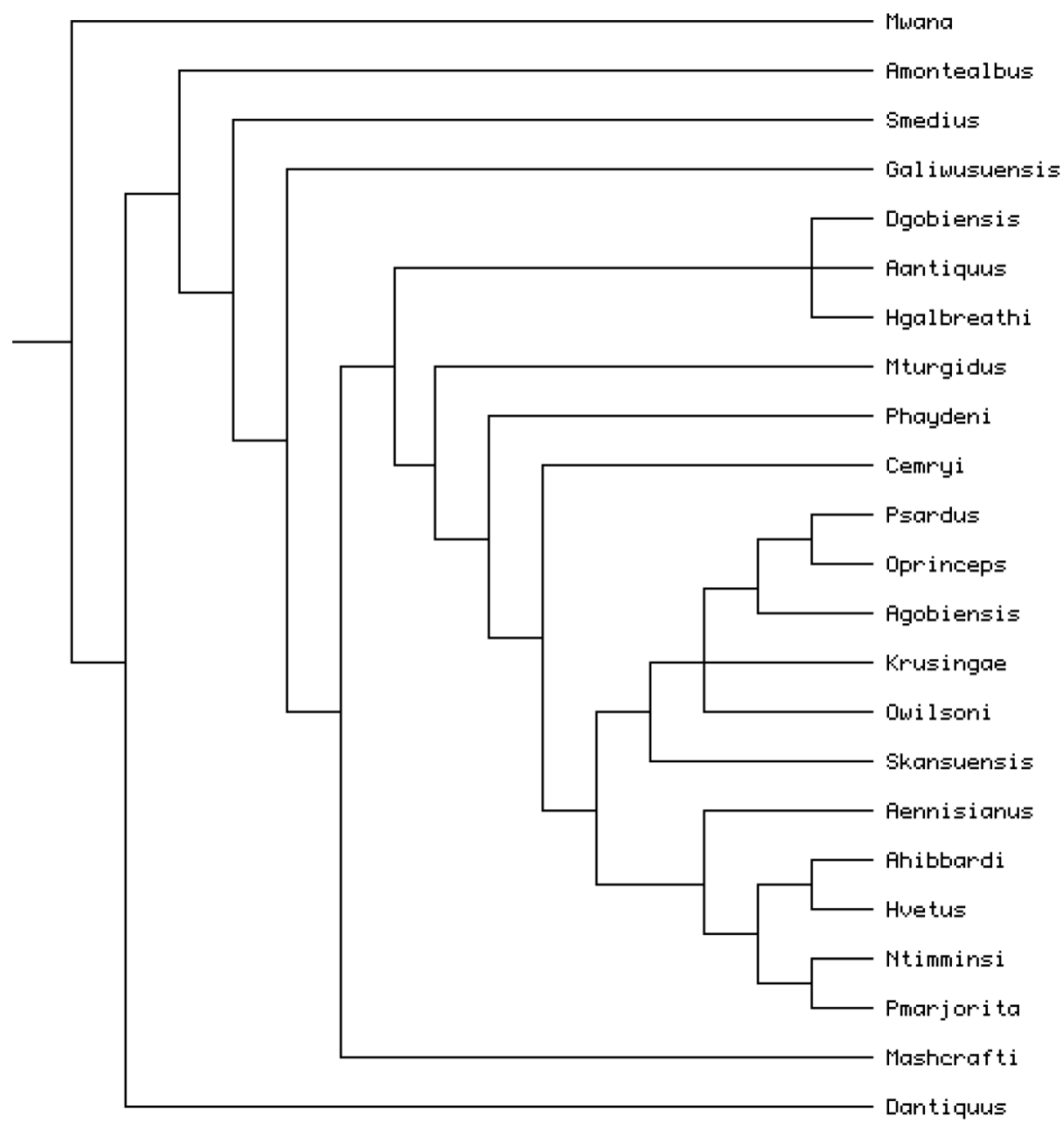

Supplement: Tree S1 — The strict consensus of 4 equally most parsimonious trees (MPT). Tree length (TL) = 280, consistency index (CI) = 0.5714, retention index (RI) = 0.7345. 22 lagomorph taxa are included; Mimotona wana, a duplicidentate Glires representative is an outgroup. The data matrix was subjected to heuristic parsimony searches with TBR branch swapping algorithm and at least 5 000 random addition replicates in PAUP* version 4.0b10 [28]. All characters unordered and unweighted and the delayed transformation (DELTRAN) optimization was used. (PDF) [file pone.0079794.s003.pdf]
